# Functional characterization of the ribosome biogenesis factors PES, BOP1, and WDR12 (PeBoW), and mechanisms of defective cell growth and proliferation caused by PeBoW deficiency in Arabidopsis

**DOI:** 10.1093/jxb/erw288

**Published:** 2016-07-20

**Authors:** Chang Sook Ahn, Hui Kyung Cho, Du-Hwa Lee, Hee-Jung Sim, Sang-Gyu Kim, Hyun-Sook Pai

**Affiliations:** ^1^Department of Systems Biology, Yonsei University, Seoul 120–749, Korea; ^2^Center for Genome Engineering, Institute for Basic Science, Daejeon 305–811, Korea

**Keywords:** Cell cycle genes, growth defects, kinematic analysis, nucleolar stress, phytohormones, ribosome biogenesis.

## Abstract

The nucleolar protein pescadillo (PES) controls biogenesis of the 60S ribosomal subunit through functional interactions with Block of Proliferation 1 (BOP1) and WD Repeat Domain 12 (WDR12) in plants. In this study, we determined protein characteristics and *in planta* functions of BOP1 and WDR12, and characterized defects in plant cell growth and proliferation caused by a deficiency of PeBoW (PES-BOP1-WDR12) proteins. Dexamethasone-inducible RNAi of *BOP1* and *WDR12* caused developmental arrest and premature senescence in Arabidopsis, similar to the phenotype of *PES* RNAi. Both the N-terminal domain and WD40 repeats of BOP1 and WDR12 were critical for specific associations with 60S/80S ribosomes. In response to nucleolar stress or DNA damage, PeBoW proteins moved from the nucleolus to the nucleoplasm. Kinematic analyses of leaf growth revealed that depletion of PeBoW proteins led to dramatically suppressed cell proliferation, cell expansion, and epidermal pavement cell differentiation. A deficiency in PeBoW proteins resulted in reduced cyclin-dependent kinase Type A activity, causing reduced phosphorylation of histone H1 and retinoblastoma-related (RBR) protein. *PeBoW* silencing caused rapid transcriptional modulation of cell-cycle genes, including reduction of *E2Fa* and Cyclin D family genes, and induction of several *KRP* genes, accompanied by down-regulation of auxin-related genes and up-regulation of jasmonic acid-related genes. Taken together, these results suggest that the PeBoW proteins involved in ribosome biogenesis play a critical role in plant cell growth and survival, and their depletion leads to inhibition of cell-cycle progression, possibly modulated by phytohormone signaling.

## Highlight

PES, BOP1, and WDR12 (PeBoW) are plant ribosome biogenesis factors. *PeBoW* silencing in Arabidopsis causes immediate inhibition of leaf cell growth and proliferation through transcriptional modulation of cell-cycle genes and phytohormone-related genes.

## Introduction

Ribosome biogenesis is a fundamental process that is tightly co-ordinated with cell growth and proliferation. Assembly of a functional ribosome is a complex, multi-step process with high demands in energy and resources (Henras *et al.*, 2008; Kressler *et al.*, 2010; Panse and Johnson, 2010; Karbstein, 2011; Woolford and Baserga, 2013). The mechanisms of rRNA processing and ribosome biogenesis have been elucidated in yeast, but not in higher eukaryotes including plants, due to their greater complexity. Orthologs of approximately 75% of yeast ribosome assembly factors have been identified in plants (Ebersberger *et al.*, 2014), but only a small fraction of these factors have been functionally characterized (Pestov *et al.*, 2001; Brown and Shaw, 2008; Horiguchi *et al.*, 2012; Cho *et al.*, 2013; Missbach *et al.*, 2013; Weis *et al.*, 2014; Jeon *et al.*, 2015). Furthermore, it is largely unknown how plant ribosome biogenesis is regulated under fluctuating metabolic and environmental conditions.

Although the nucleolus is primarily associated with ribosome biogenesis, recent evidence suggests its involvement in diverse cellular processes, such as cell-cycle control, stress sensing and responses, DNA damage repair, pre-mRNA processing, and telomere metabolism (Boisvert *et al.*, 2007; Shaw and Brown, 2012; Lam and Trinkle-Mulcahy, 2015). Perturbations in ribosome biogenesis or function in mammalian cells leads to nucleolar stress, which can cause cell-cycle arrest, senescence, and apoptosis through activation of the tumor suppressor p53 (Antoniali *et al.*, 2014; Golomb *et al.*, 2014; James *et al.*, 2014). In this pathway, the ribosomal proteins RPL5 and RPL11 diffuse from the nucleolus to the nucleoplasm following nucleolar stress and bind to the E3 ubiquitin ligase MDM2, thereby blocking MDM2-mediated ubiquitination and degradation of p53, ultimately leading to cell-cycle arrest. However, nucleolar stress results in arrested cell proliferation in organisms lacking p53, such as yeast, *Drosophila*, and plants, suggesting a link between p53-independent mechanisms for nucleolar stress and the cell cycle (Donati *et al.*, 2012; Cho *et al.*, 2013; James *et al.*, 2014).

Pescadillo (PES) is an evolutionarily conserved nucleolar protein that is essential for the viability of yeast and higher eukaryotes (Allende *et al.*, 1996; Adams *et al.*, 2002; Lerch-Gaggl *et al.*, 2002). PES associates with Block of Proliferation 1 (BOP1) and WD Repeat Domain 12 (WDR12) to form the PeBoW complex in mammalian cells, and together they modulate pre-rRNA processing for the synthesis of 28S and 5.8S rRNAs, failure of which causes defective assembly of the 60S large ribosomal subunit (Strezoska *et al.*, 2000; Lapik *et al.*, 2004; Hölzel *et al.*, 2005; Grimm *et al.*, 2006; Rohrmoser *et al.*, 2007). Yeast counterparts of these three proteins, Nop7/Yph1 (PES), Erb1 (BOP1), and Ytm1 (WDR12), form the Nop7 subcomplex in yeast pre-ribosomes, which are required to process 27S pre-rRNA into mature 25S and 5.8S rRNAs (Adams *et al.*, 2002; Miles *et al.*, 2005). Down-regulation or expression of a dominant negative mutant of PES, BOP1, or WDR12 results in cell-cycle arrest with altered expression of cell-cycle regulators, suggesting their function in cell proliferation control (Pestov *et al.*, 2001; Hölzel *et al.*, 2005; Li *et al.*, 2009). Misregulation of *PES* and *BOP1* has been linked to chromosomal instability and tumourigenesis (Killian *et al.*, 2004; Li *et al.*, 2009; Chung *et al.*, 2011).

Our previous studies have shown that the PeBoW (PES-BOP1-WDR12) paradigm observed in mammals and yeast appears to be conserved in plants (Cho *et al.*, 2013). Plant PES was found to interact with BOP1 and WDR12 in the nucleolus. Silencing of Arabidopsis *PES* via dexamethasone (DEX)-inducible RNAi delayed maturation of 25S rRNA and suppressed global translation, causing growth arrest and acute cell death. Virus-induced gene silencing of any of the *PeBoW* genes in *Nicotiana benthamiana* resulted in defective biogenesis of the 60S large ribosomal subunit. These results suggest that PES is essential to plant cell growth and survival by modulating ribosome biogenesis through a functional link with BOP1 and WDR12 (Cho *et al.*, 2013). In this current study, we investigated further the protein characteristics of PES, BOP1, and WDR12, and analyzed phenotypes of *BOP1*- and *WDR12*-silenced Arabidopsis plants. In addition, we explored the molecular mechanisms of cell-cycle inhibition by disrupted ribosome biogenesis in PeBoW-deficient Arabidopsis plants.

## Materials and methods

### Plant materials and growth conditions

*Arabidopsis thaliana* (ecotype Columbia-0) and *Nicotiana benthamiana* plants were grown in soil in a growth chamber at 22 °C under a 16-h light/8-h dark cycle. For growth on agar, Arabidopsis seeds were surface-sterilized and sown on Petri dishes containing MS medium [Murashige and Skoog salts (pH 5.7), 0.35% Phytagel (Sigma), and 2% sucrose] with ethanol (–DEX) or 10 µM DEX. For liquid culture, plant seedlings were grown in six-well plates containing 1ml of liquid medium [0.5 × Murashige and Skoog salts (pH 5.7) and 0.5% sucrose] at 23 °C and 100–120 μmol m^−2^ s^−1^ light intensity under a 16h light/8h dark cycle. At 7 d after sowing, seedlings were treated with ethanol or 20 μM DEX for 12h or 24h.

### Generation of dexamethasone (DEX)-inducible *BOP1* and *WDR12* RNAi lines in Arabidopsis

For *BOP1* RNAi, a 291-bp *BOP1* cDNA fragment was PCR-amplified using BOP1-sense (F)/(R) primers containing XhoI and ClaI sites (5′-gctccacatgcggactttga-3′ and 5′-tcctggcaatttaagcttggg-3′) for the sense construct and BOP1-antisense (F)/(R) primers containing SpeI and BamHI sites (5′-gctccacatgcggactttga-3′ and 5′-tcctggcaatttaagcttggg-3′ for the antisense construct. For *WDR12* RNAi, a 300-bp *WDR12* cDNA fragment was PCR-amplified using WDR12-sense (F)/(R) primers containing XhoI and ClaI sites (5′-atggatatcgacggagaaga-3′ and 5′-tggtgtcacagcccttatgt-3′) for the sense construct and WDR12-antisense (F)/(R) primers containing SpeI and BamHI sites (5′-atggatatcgacggagaagatgtat-3′ and 5′-tggtgtcacagccct-3′) for the antisense construct. Using these constructs, DEX-inducible *BOP1* and *WDR12* RNAi transgenic Arabidopsis lines (ecotype Columbia-0) were generated by a floral dip method. After floral-dipping, seeds were harvested and sown on MS medium containing hygromycin (30mg l^–1^). Hygromycin-resistant primary T_1_ transformants were moved to soil to grow and set seeds. Seeds obtained from each T_1_ transformant were grown on hygromycin-containing medium to calculate the ratio of hygromycin-resistant to hygromycin-sensitive seedlings. Only the lines showing 3:1 segregation ratio were selected (T_2_ generation), and tested for gene-silencing phenotypes by germinating the seeds in ethanol- or DEX-containing medium. Dexamethasone (Sigma) was added to the medium to a final concentration of 10 μM in ethanol (0.033%) from 30mM stock solution. Five-to-eight independent T_2_ lines that showed strong silencing phenotypes on DEX-containing medium were selected for T_3_ propagation. Ten-to-sixteen plants of each selected independent T_2_ line were grown in soil to obtain seeds, and the seed batch that showed 100% hygromycin-resistance was selected as the homozygous T_3_ generation. For RNAi of *BOP1* and *WDR12*, more than 30 independent T_2_ lines each were generated. Among the T_2_ lines tested, six independent *BOP1* RNAi lines and five independent *WDR12* RNAi lines exhibited strong growth retardation phenotypes when grown on DEX-containing medium, and were subsequently propagated for the T_3_ generation. Two independent *BOP1* and *WDR12* lines were finally selected, and their T_3_ and T_4_ homozygous seeds were used for the analyses throughout the study.

### Agrobacterium-mediated transient expression

Agroinfiltration was carried out as described previously by Cho *et al.* (2013).

### Subcellular localization using GFP fusion

Green fluorescent protein (GFP) fusion and confocal microscopy were performed as described previously by Cho *et al.* (2013). For drug treatment, GFP-fused PeBoW proteins were expressed in *N. benthamiana* leaves via agroinfiltration. After 36h, the leaves were treated with 20 μM mycophenolic acid (MPA), 5 μM Actinomycin D, and 0.03% methyl methane sulfate (MMS) by syringe infiltration. Epidermal cells of the leaves were then periodically observed directly by confocal microscopy.

### Kinematic analysis of leaf growth

Kinematic analysis was performed as described previously by De Veylder *et al.* (2001) and Ahn *et al.* (2015).

### RNA extraction from seedlings

*PES*, *BOP1*, and *WDR12* RNAi seedlings were grown on MS media for 7 d and then transferred to medium containing 10 µM DEX. At 2 and 4 d after transfer, total RNA was isolated from equal fresh weights of seedlings using the IQeasy^TM^ plus plant RNA extraction kit (iNtRON Biotechnology, Korea) according to the manufacturer’s instructions. The concentration and purity of RNAs were determined by NanoDrop 1000 (Thermo Scientific).

### Real-time quantitative RT-PCR

Real-time quantitative RT-PCR was performed using gene-specific primers as described previously by Cho *et al.* (2013). Supplementary Table S1 at *JXB* online lists the primers used in this study.

### Immunoblotting

Immunoblotting was performed as described by Ahn *et al.* (2011) and Cho *et al.* (2013), using mouse monoclonal antibodies against the Flag tag (Sigma), GFP (Clontech), RPL10a (Santa Cruz Biotechnology), and histone H3 (Santa Cruz Biotechnology). The immunoblots were treated with horseradish peroxidase-conjugated goat anti-mouse IgG antibodies (Invitrogen), followed by signal detection using Imagequant LAS 4000 (GE Healthcare Life Sciences).

### Co-immunoprecipitation

Co-immunoprecipitation was performed as described previously by Ahn *et al.* (2011) and Cho *et al.* (2013).

### Measurement of cyclin-dependent kinase (CDK) activity

To purify glutathione S-transferase (GST)-fusion protein of the C-terminal domain of Arabidopsis RBR (GST-RBR-C), the *RBR* cDNA fragment corresponding to the amino acid residues 858–1014 was amplified by PCR and cloned into the pGEX-4T-1 vector (Amersham Bioscience). GST-RBR-C proteins were purified using Glutathione Excellose resin (Amersham Bioscience) following the manufacturer’s instructions. The p13^Suc1^-associated CDK activity was assayed as described by Boniotti and Gutierrez (2001) using histone H1 (Millipore) or the purified recombinant GST-RBR-C proteins as substrates.

### Sucrose gradient sedimentation

Sucrose density gradient centrifugation was performed according to Cho *et al.* (2013).

### Quantitative analysis of endogenous jasmonic acid and abscisic acid content

Frozen samples (0.15–0.17g) were grinded and phytohormones were extracted with 1ml of ethyl acetate (Lee *et al.*, 2015). Ultra-performance liquid chromatography (ACQUITY®UPLC system, Waters Corp., Milford, MA, USA) coupled with a QTOF instrument (XEVO G2XS; Waters Corp.) was used for the analysis. The chromatographic separation was performed on an ACQUITY®UPLC BEH C18 column (100×2.1mm, i.d. 1.7 μm). The mobile phases consisted of solvent A (0.1% formic acid) and solvent B (acetonitrile). The gradient elution mode was programmed as follows: 20–25% B for 0.0–5.0min and 25–35% B for 5.0–10.0min. Mass spectrometry analysis was conducted in the negative ion mode with electrospray ionization (ESI).

### Statistical analyses

Two-tailed Student’s *t*-tests were performed using the Minitab 16 program (Minitab Inc.; http://www.minitab.com) to investigate the statistical differences between the responses of the samples. Significant differences between controls and other samples are indicated as follows: *P≤0.05 and **P≤0.01.

## Results

### Characterization of dexamethasone (DEX)-inducible *BOP1* and *WDR12* RNAi lines

**Fig. 1.**
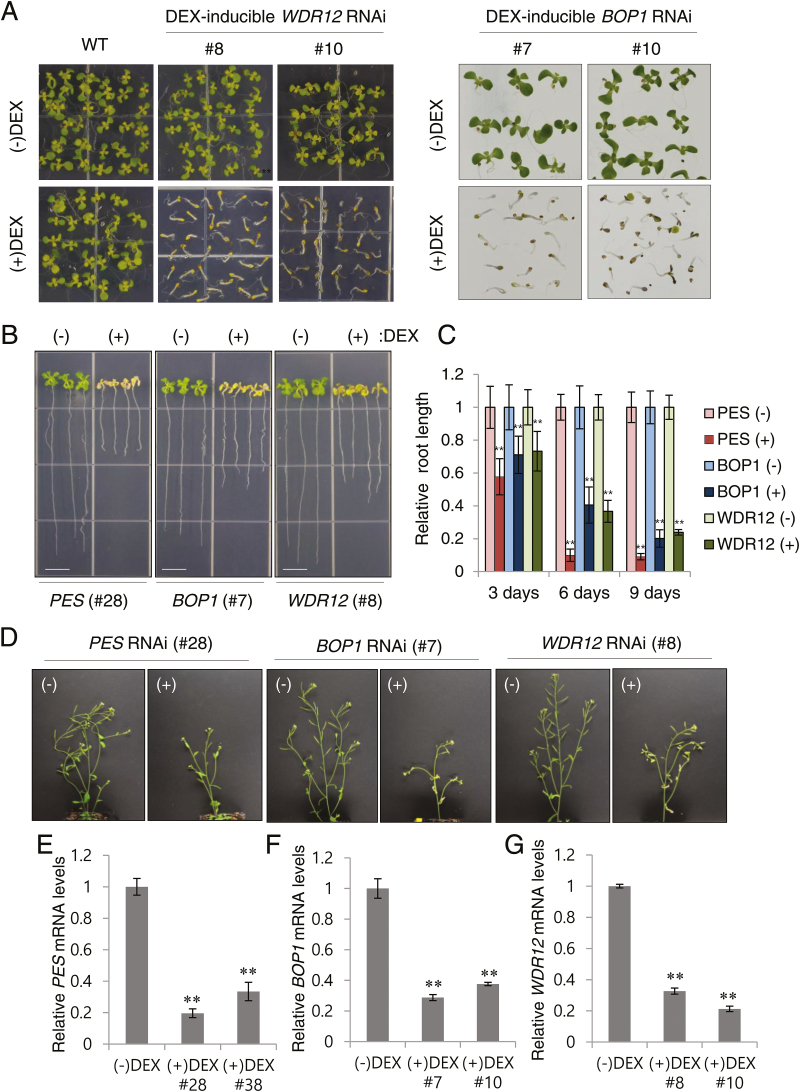
Growth arrest and premature senescence phenotypes of *BOP1*- and *WDR12*-silenced plants. (A) Growth arrest phenotype of Arabidopsis dexamethasone (DEX)-inducible *BOP1* (#7 and #10) and *WDR12* (#8 and #10) RNAi lines. Seedlings were germinated on MS media that contained either ethanol (–DEX) or 10 μM DEX. (B) Retarded root growth and premature senescence of aerial tissues. DEX-inducible *PES* (#28), *BOP1* (#7), and *WDR12* (#8) RNAi seedlings were grown on MS media and then transferred to (–)DEX or (+)DEX media for vertical growth. Scale bars are 9mm. (C) Root length measurements in RNAi seedlings 3, 6, and 9 d after transfer to (–)DEX or (+)DEX media. Each data point represents the mean ± SD (*n*=20). Asterisks denote statistical significance as follows: *, *P*≤0.05; **, *P*≤0.01. (D) Premature senescence of the inflorescence in DEX-treated RNAi plants. (E–G) Relative transcript levels in RNAi lines. Real-time quantitative RT-PCR analyses were performed on two independent *PES* (E), *BOP1* (F), and *WDR12* (G) RNAi lines. Transcript levels were quantified relative to (–)DEX samples using *UBC10* mRNA levels as a control. Each value represents the mean ±SD of three replicates per experiment. (This figure is available in colour at *JXB* online.)

We generated DEX-inducible RNAi lines of *BOP1* and *WDR12* in Arabidopsis, in addition to the *PES* RNAi lines generated previously (Cho *et al.*, 2013). Transgenic Arabidopsis plants (Col-0 ecotype) carried either a *BOP1* RNAi construct that had an inverted repeat of a 291-bp *BOP1* cDNA fragment or a *WDR12* RNAi construct that had an inverted repeat of a 300-bp *WDR12* cDNA fragment, both of which were under the control of a DEX-inducible transcription system (Aoyama and Chua, 1997). When sown on media containing 10 µM DEX, shoot growth of two independent *PES* (#28 and #38), *BOP1* (#7 and #10) and *WDR12* (#8 and #10) RNAi lines was immediately arrested following germination and plants died prematurely, mostly without forming true leaves, whereas seedlings grew normally on ethanol-containing medium (–DEX) (Fig. 1A and Supplementary Fig. S1; Cho *et al.*, 2013). When these RNAi seedlings were grown on MS media for 7 d and then transferred to media containing 10 µM DEX for additional growth for 3–9 d, they exhibited precocious senescence of aerial tissues, anthocyanin accumulation, and retarded root growth compared with the control treatment (–DEX) (Fig. 1B, C; Supplementary Fig. S2A). These results suggest that *PES*, *BOP1*, and *WDR12* play a critical role in early plant growth and development. When RNAi plants were grown in soil for 3 weeks and then sprayed with ethanol (–DEX) or 30 µM DEX for 5 d, DEX inhibited further development of inflorescences and induced senescence of flowers, cauline leaves, and siliques (Fig. 1D). The effect of RNAi on *PES*, *BOP1*, and *WDR12* mRNA levels in the independent RNAi seedlings was determined by real-time quantitative RT-PCR using gene-specific primers (Fig. 1E–G; Supplementary Table S1). After transfer to media containing 10 µM DEX for additional growth for 3 d, seedlings of the corresponding RNAi lines exhibited significantly reduced *PES*, *BOP1*, and *WDR12* transcript levels as compared to (–)DEX samples, suggesting RNAi-induced gene silencing. DEX treatment for 4 d, but not for 2 d, resulted in a decrease in total cellular 25S and 18S rRNAs in *BOP1* (#7 and #10) and *WDR12* (#8 and #10) RNAi lines, suggesting rRNA degradation (see Supplementary Fig. S3), as previously observed in *PES* RNAi lines (Cho *et al.*, 2013).

### Subcellular localization of Arabidopsis BOP1, WDR12, and their mutants

**Fig. 2.**
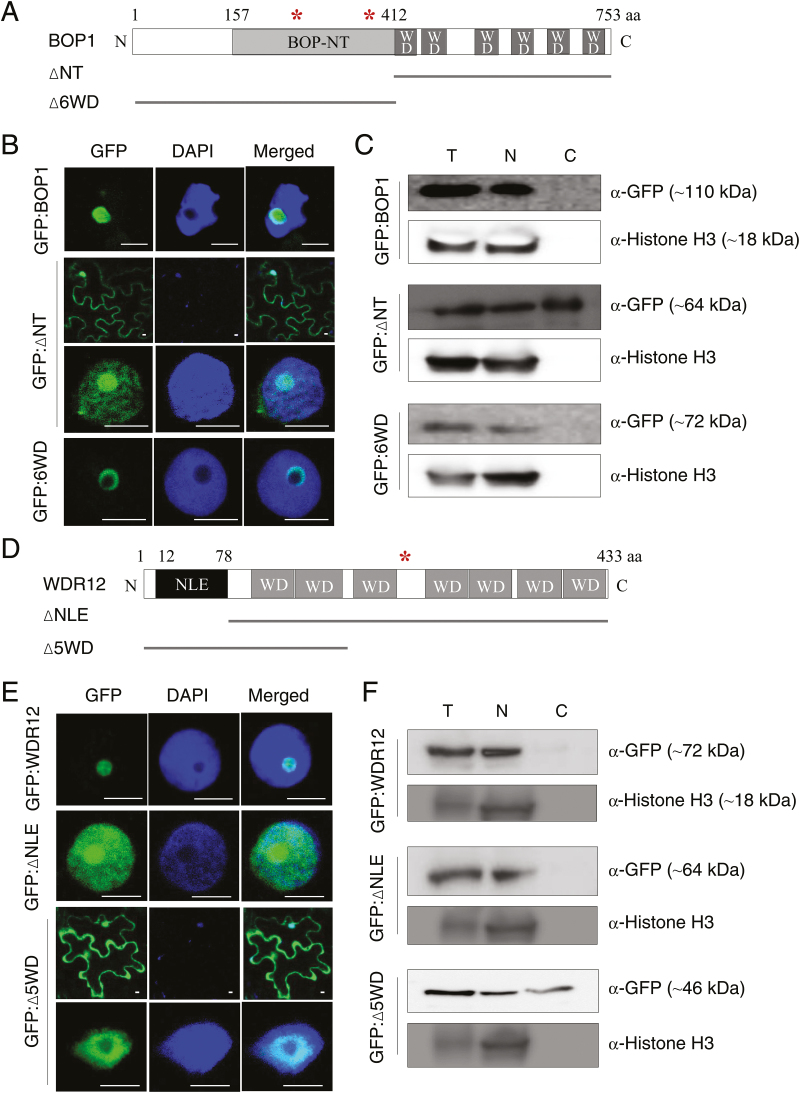
Subcellular localization of BOP1, WDR12, and their mutants. (A) Schematic of BOP1 and its deletion mutants (∆NT and ∆6WD). BOP-NT, BOP1 N-terminal domain; WD, WD40 domain. Nuclear localization signals (asterisks) are marked at amino acid residues 253 and 386. aa, amino acids. (B) Subcellular localization of BOP1 and its mutants using GFP fusion. The infiltrated leaves were briefly stained with DAPI to mark nuclei and examined by confocal laser scanning microscopy. Scale bars are 5 µm. (C) Subcellular fractionation. *Nicotiana benthamiana* leaf extracts expressing GFP fusion proteins of BOP1 and its mutants were fractionated and subjected to immunoblotting with anti-GFP antibodies. Total (T), nuclear (N), and cytosolic (C) fractions are indicated. Histone H3 was detected as a nuclear marker protein. The sizes of the protein bands are indicated. (D) Schematic of WDR12 and its deletion mutants (∆NLE and ∆5WD). NLE, Notchless-like domain; WD, WD40 domain. An asterisk at residue 246 indicates the nuclear localization signal. (E) Subcellular localization of WDR12 and its mutants using GFP fusion. Scale bars are 5 µm. (F) Leaf extracts expressing GFP fusion proteins of WDR12 and its mutants were fractionated and subjected to immunoblotting as described in (C). The sizes of the protein bands are indicated. (This figure is available in colour at *JXB* online.)

Previously, we have shown that BOP1 and WDR12 are mainly localized in the nucleolus (Cho *et al.*, 2013). To determine which protein domains contribute to nucleolar localization, GFP fusion proteins of BOP1 and WDR12 deletion mutants were expressed in *N. benthamiana* leaves via agroinfiltration. BOP1 contains the BOP1 N-terminal domain (BOP-NT), six WD40 domains in the C-terminus, and two nuclear localization signals (marked with asterisks in Fig. 2A). Confocal laser scanning microscopy of leaf epidermal cells revealed that GFP:BOP1 predominantly localized in the nucleolus. However, deletion of the N-terminal region (∆NT), including the BOP-NT domain, resulted in BOP1 distribution in the nucleus and the cytosol, suggesting the importance of the N-terminal region for BOP1 nucleolar localization (Fig. 2B). When the C-terminal region containing six WD40 domains was deleted (∆6WD), GFP fluorescence was mostly detected in the nucleolar periphery (Fig. 2B). To confirm the subcellular localization of the BOP1 mutants, total (T), nuclear (N), and cytosolic (C) protein fractions were prepared from the agroinfiltrated *N. benthamiana* leaves, and subjected to immunoblot analyses using anti-GFP antibodies and anti-histone H3 antibodies as a nuclear marker (Fig. 2C). GFP:BOP1 and GFP:∆6WD were mostly associated with nuclear fractions, whereas GFP:∆NT was present in both nuclear and cytosolic fractions, consistent with the confocal data (Fig. 2B).

WDR12 contains the Notchless-like domain (NLE) at its N-terminus, followed by seven WD40 domains, and a nuclear localization signal at its center (Fig. 2D). In contrast to the predominant nucleolar localization of GFP:WDR12, GFP:∆NLE lacking the NLE domain was distributed throughout the nucleus (Fig. 2E). Green fluorescence of GFP:∆5WD lacking five C-terminal WD40 domains was observed in both the nucleus and the cytosol. The ∆7WD mutant that lacks all of the seven WD40 domains was not stably expressed in *N. benthamiana* leaves, regardless of the position of GFP tagging. These results suggest that both the NLE and C-terminal WD40 domains function in nucleolar localization of WDR12. Immunoblotting using anti-GFP antibodies detected GFP:∆NLE in the nuclear fraction, and GFP:∆5WD in both nuclear and cytosolic fractions (Fig. 2F), supporting the results of confocal microscopy (Fig. 2E).

### *In vivo* interactions and ribosome association of the BOP1 and WDR12 mutants

**Fig. 3.**
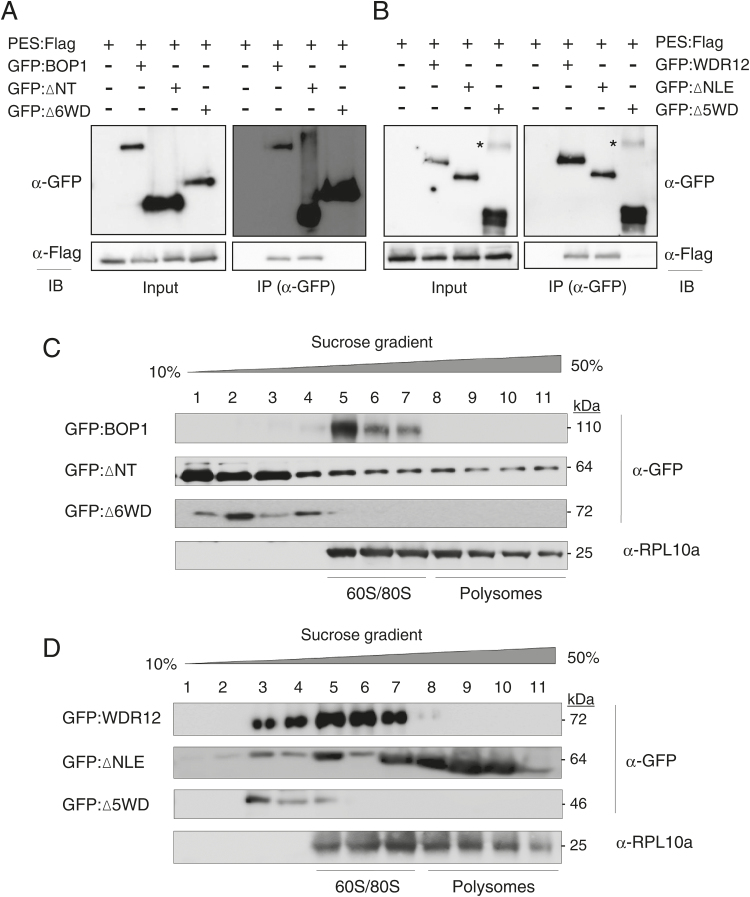
Protein interactions and co-fractionation with ribosome subunits. (A) Co-immunoprecipitation of BOP1 with PES. Protein extracts were subjected to immunoprecipitation (IP) with anti-GFP antibody, and then co-immunoprecipitated PES:Flag was detected by immunoblotting (IB) with anti-Flag antibody. The asterisks indicate non-specific protein bands. (B) Co-immunoprecipitation of WDR12 with PES. Co-immunoprecipitation was performed as described in (A). (C) Co-fractionation of BOP1 and its mutants with ribosome subunits. After sedimentation of ribosomes through a sucrose density gradient, the fractions were subjected to immunoblotting with anti-GFP and anti-ribosomal protein L10a (RPL10a) antibodies. Lanes 1–11 indicate gradient fractions from top (10%) to bottom (50%). (D) Co-fractionation of WDR12 and its mutants with ribosome subunits. Sucrose density gradient centrifugation and immunoblotting were performed as described in (C).

To demonstrate *in vivo* protein interactions between PES and deletion mutants of BOP1 and WDR12, co-immunoprecipitation was performed. Flag-fused PES (PES:Flag) was expressed together with GFP-fused BOP1, ∆NT, or ∆6WD in *N. benthamiana* leaves by agroinfiltration (Fig. 3A). Protein expression was confirmed by immunoblotting with anti-Flag and anti-GFP antibodies (input). GFP-fusion proteins were immunoprecipitated from cell extracts of the infiltrated leaves with anti-GFP antibodies. Immunoblotting was performed with anti-GFP antibodies to detect immunoprecipitated GFP-fusion proteins, and then with anti-Flag antibodies to detect PES:Flag as co-immunoprecipitants. GFP:BOP1 and GFP:∆NT were co-immunoprecipitated with PES:Flag, but co-immunoprecipitation did not take place when PES:Flag was expressed alone or co-expressed with GFP:∆6WD (Fig. 3A and Supplementary Fig. S4A). These results suggest that the six C-terminal WD domains are important for BOP1 interaction with PES (Fig. 3A). Similarly, GFP:WDR12 and GFP:∆NLE, but not GFP:∆5WD, were co-immunoprecipitated with PES:Flag, suggesting that the five C-terminal WD domains are important for WDR12 interaction with PES (Fig. 3B and Supplementary Fig. S4B).

To investigate which protein domains are important for the association of BOP1 and WDR12 with ribosomes, leaf cell extracts expressing GFP-fusion proteins of BOP1, WDR12, and their deletion mutants were fractionated on a 10–50% sucrose density gradient. After ultracentrifugation, fractions were collected, and immunoblot analysis was performed with anti-GFP antibodies (Fig. 3C, D). As a control, the same fractions were reacted with anti-RPL10a antibodies to detect the ribosomal protein L10a associated with 60S large ribosomal subunits, 80S monosomes, and polysomes. Full-length BOP1 was co-fractionated with the 60S/80S ribosome, whereas the ∆6WD mutant was detected in the lighter fractions of the gradient (Fig. 3C). ∆NT lacking the BOP1 N-terminus was detected in every fraction throughout the gradient, suggesting a lack of interaction specificity (Fig. 3C). WDR12 was mainly detected in fractions containing the 60S/80S ribosome and partially in the lighter fractions, whereas the ∆5WD mutant was detected only in the lighter fractions (Fig. 3D). ∆NLE was detected in most fractions of the gradient, lacking interaction specificity. Collectively, these results suggest that both the N-terminal domains and C-terminal WD40 domains are required for the specific association of BOP1 and WDR12 with 60S/80S ribosomes.

### Translocation of PES, BOP1, and WDR12 into the nucleoplasm upon drug treatment

**Fig. 4.**
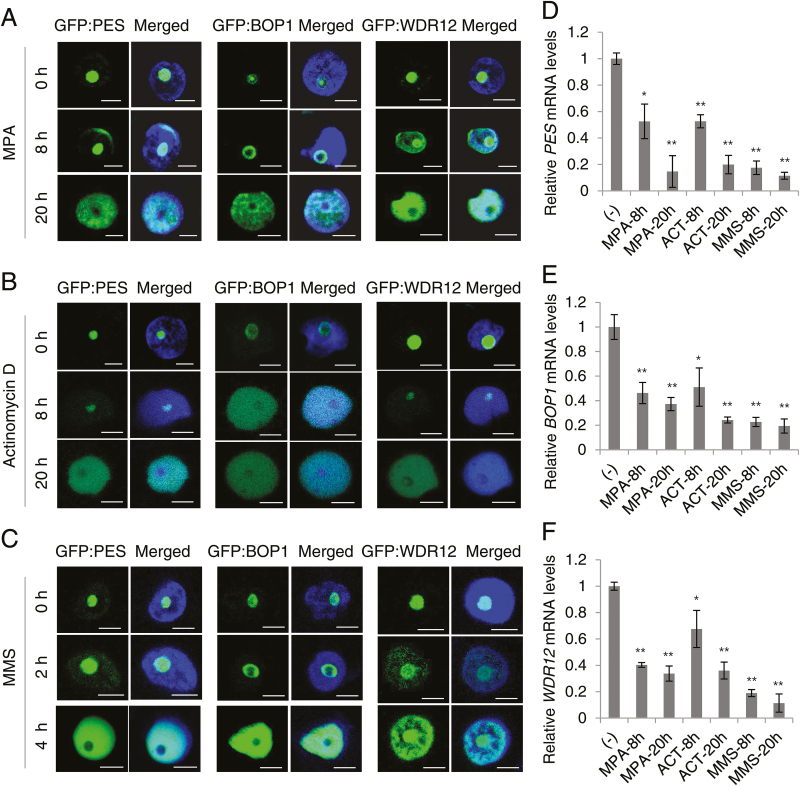
Translocation of PeBoW proteins into the nucleoplasm in response to drug treatment. (A) Effects of mycophenolic acid (MPA). *Nicotiana benthamiana* leaves were agroinfiltrated with GFP fusion constructs and then treated with MPA (20 µM) for 0, 8, and 20h. Merged images with DAPI-stained nuclei are shown. Scale bars are 5 µm. (B) Effects of actinomycin-D (5 µM) after 0, 8, and 20h treatment. (C) Effects of methyl methanesulfonate (MMS; 0.03%) after 0, 2, and 4h treatment. (D–F) Real-time quantitative RT-PCR. Transcript levels of *PES* (D), *BOP1* (E), and *WDR12* (F) after drug treatment were compared with those prior to treatment (–). The *UBC10* mRNA level was used as a control. (This figure is available in colour at *JXB* online.)

Treatment of animal and plant cells with a drug that depletes cellular guanine nucleotides or disrupts pre-rRNA synthesis leads to nucleolar disruption and an efflux of nucleolar proteins, such as nucleostemin, nucleolin, and nucleophosmin, into the nucleoplasm (Mayer and Grummt, 2005; Tsai and McKay, 2005; Boisvert *et al.*, 2007; Jeon *et al.*, 2015). We tested if nucleolar localization of PeBoW proteins is affected by these drugs. *Nicotiana benthamiana* leaves were agroinfiltrated to express GFP fusion proteins of PES, BOP1, and WDR12, and then treated with mycophenolic acid (MPA), actinomycin-D, or methyl methanesulfonate (MMS). MPA inhibits *de novo* synthesis of guanine nucleotides, subsequently disrupting rRNA synthesis and inducing nucleolar stress in human cells (Tsai and McKay, 2005; Huang *et al.*, 2008). A low concentration of actinomycin-D specifically inhibits RNA polymerase I and blocks rRNA transcription (Tsai and McKay, 2005). MMS damages RNA as well as DNA by alkylation, and damaged rRNA molecules can lead to ribosomal dysfunction (Revenkova *et al.*, 1999; Lundin *et al.*, 2005). Confocal microscopy revealed that PES, BOP1, and WDR12 migrated from the nucleolus to the nucleoplasm 8 to 20h post-treatment with MPA or actinomycin-D (Fig. 4A, B). Translocation occurred even faster after MMS treatment, taking place within 4h (Fig. 4C). These results suggest that disrupted rRNA synthesis and nucleolar stress caused repartitioning of PeBoW proteins into the nucleoplasm. Real-time quantitative RT-PCR analyses revealed that transcript levels of *PES*, *BOP1*, and *WDR12* all decreased upon drug treatment (Fig. 4D–F). Although the mechanism of protein translocation is unclear, efflux of biogenesis factors, such as PES, BOP1, and WDR12, may represent a cellular response to stresses by curtailing ribosome biogenesis, an extremely resource-demanding process.

### Kinematic analyses of leaf growth

**Fig. 5.**
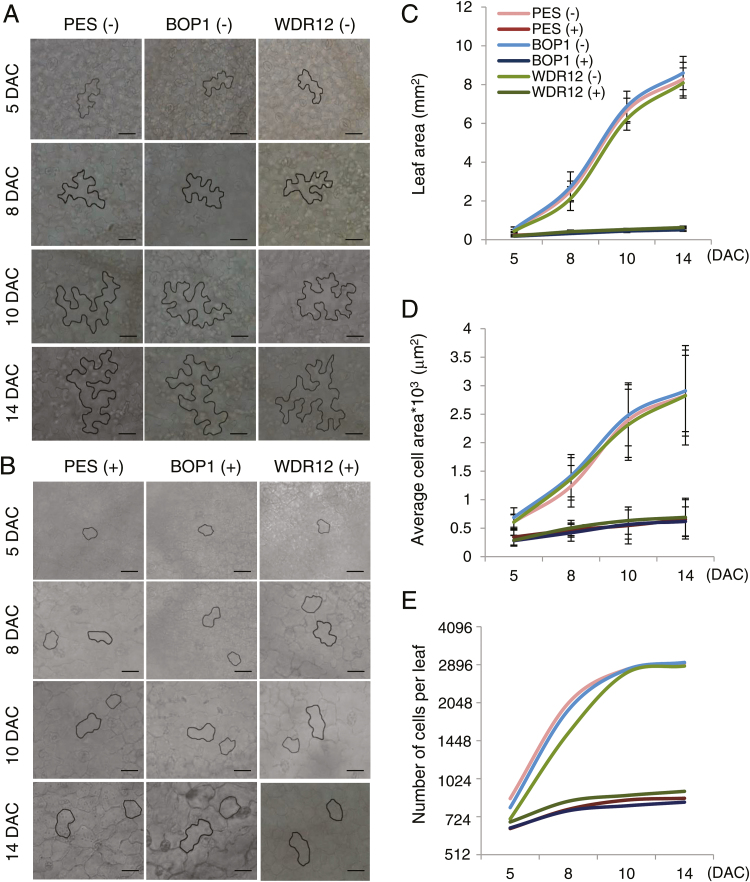
Kinematic analysis of leaf growth. *PES* (#28), *BOP1* (#7), and *WDR12* (#8) RNAi plants were grown in soil and sprayed with ethanol (–) or 20 µM DEX (+). The first leaves were collected from the plants at 5, 8, 10, and 14 d after cotyledon emergence (DAC). (A, B) Representative abaxial epidermal cells from the leaves of RNAi seedlings sprayed with ethanol (A) or DEX (B). Individual cells are visualized by black outlines using ImageJ. Scale bars are 20 µm. (C) Average leaf area. (D) Average leaf epidermal cell area. (E) Calculated number of epidermal cells per leaf. (This figure is available in colour at *JXB* online.)

Cell division and cell expansion are two parameters of organ growth (Horiguchi *et al.*, 2006; Anastasiou and Lenhard, 2007). To determine the effect of PeBoW depletion on cell division and expansion during leaf development, kinematic analyses were performed on the first true leaves of *PES* (#28), *BOP1* (#7), and *WDR12* (#8) RNAi plants (Fig. 5). After spraying with ethanol (–DEX) or 20 µM DEX, the first leaves of four-to-five plants were harvested at 5, 8, 10, and 14 d after cotyledon emergence (DAC) to assess leaf size and abaxial epidermal cell size, and to calculate the epidermal cell number using these two values (Fig. 5A, B). In DEX-treated plants, necrotic lesions developed in the first leaves after 14 DAC. The average leaf area and average epidermal cell area of ethanol-treated *PES*, *BOP1*, and *WDR12* RNAi lines progressively increased up to 14 DAC, whereas those values barely increased in DEX-treated RNAi plants (Fig. 5C, D and Supplementary Table S2). There was a ~14.9-fold and ~4.4-fold difference in the average leaf area and epidermal cell area between ethanol- and DEX-treated samples at 14 DAC, respectively. The epidermal cell number per leaf increased rapidly during the early stages and then remained nearly constant from 10 DAC onward for ethanol-treated RNAi plants. The cell number only slightly increased in DEX-treated plants; the estimated cell number was ~3.4-fold lower than that of ethanol-treated plants at 14 DAC (Fig. 5E and Supplementary Table S2). These results suggest that PeBoW protein depletion strongly represses both leaf cell division and expansion. Interestingly, while the epidermal pavement cells of ethanol-treated plants had a fully differentiated puzzle-shaped structure, those of DEX-treated samples exhibited a much simpler structure with little lobe formation up to 14 DAC (Fig. 5A, B). DEX treatment also caused similar defects in leaf epidermal cell growth and morphology in *PES* (#38), *BOP1* (#10), and *WDR12* (#10) RNAi lines (see Supplementary Fig. S2B). Collectively, these results suggest that defective ribosome biogenesis caused by PeBoW protein depletion inhibited cell division, cell expansion, and pavement cell differentiation.

### Reduced cyclin-dependent kinase activity in PeBoW-deficient plants

**Fig. 6.**
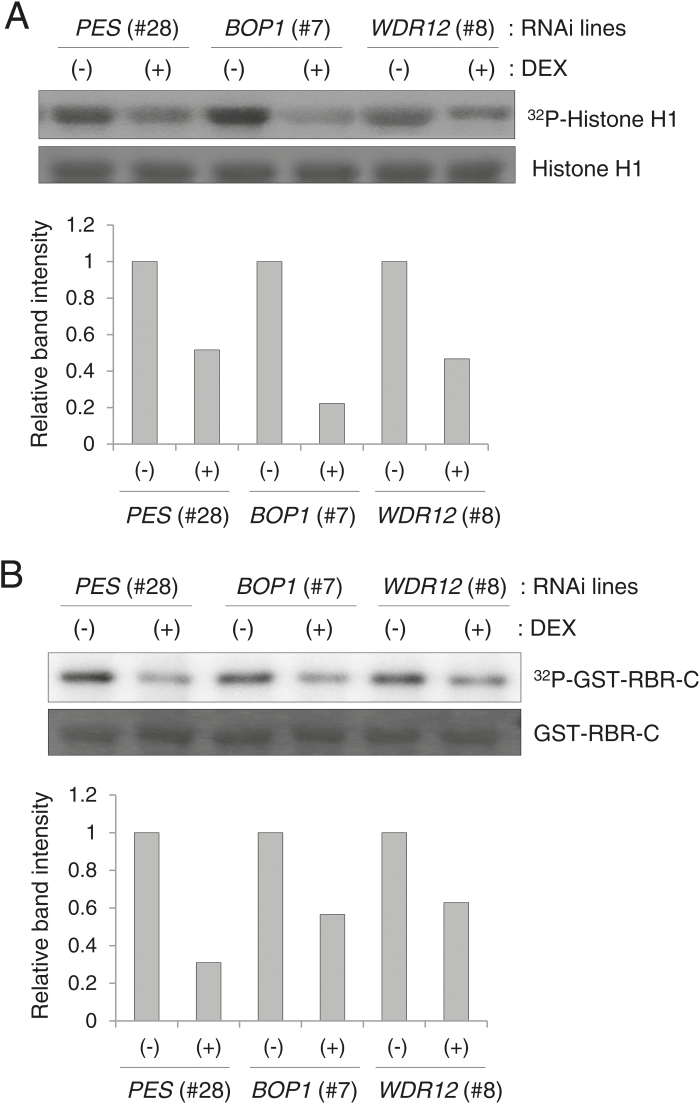
*In vitro* phosphorylation of histone H1 and the RBR C-terminus by CDKA. (A) Total CDK^PSTAIRE^ was bound to p13^Suc1^-conjugated agarose beads, and *in vitro* kinase assays were performed using histone H1 as a substrate. After SDS-PAGE, the gel was dried and analyzed with a phosphorimager to detect ^32^P-labeled histone H1, while a duplicate gel was stained with Coomassie blue to show histone H1 proteins loaded in each lane (top). Band intensities of phosphorylated histone H1 in (+)DEX samples are compared with those in (–)DEX samples (bottom). (B) *In vitro* kinase assays were performed as described in (A) using the GST fusion protein of the C-terminal domain of RBR (GST-RBR-C) as a substrate (top). Relative band intensities of phosphorylated GST-RBR-C are shown (bottom).

To investigate the molecular mechanisms of reduced cell division in *PES* (#28), *BOP1* (#7), and *WDR12* (#8) RNAi plants, we examined cyclin-dependent kinase (CDK) activity (Fig. 6). In plants, CDK Type A (CDKA), an authentic PSTAIRE CDK, plays a critical role at both the G_1_/S and G_2_/M phase transitions (Inzé and De Veylder, 2006; De Veylder *et al.*, 2007). CDKA phosphorylates histone H1 and the C-terminal domain of retinoblastoma-related (RBR) protein *in vitro* as a substrate, and CDKA activity is positively correlated with the cell division rate (Boniotti and Gutierrez, 2001). Each RNAi line was grown for 7 d in MS media, and then transferred to MS media containing ethanol (–DEX) or 10 µM DEX for further growth for 3 d. Next, protein extracts prepared from the seedlings were incubated with p13^SUC1^ beads that bind to PSTAIRE CDKs with high affinity. The glutathione S-transferase (GST) fusion protein of the C-terminal domain of Arabidopsis RBR (GST-RBR-C) was purified from *Escherichia coli*. *In vitro* kinase assays were performed with the p13^SUC1^ beads and histone H1 (Fig. 6A) or GST-RBR-C (Fig. 6B) as a substrate. Quantification of the phosphoprotein band intensities using ImageJ (http://imagej.nih.gov/ij/) revealed that the levels of phosphorylated histone H1 and GST-RBR-C were significantly reduced in (+)DEX plants, compared with those in (–)DEX plants, suggesting reduced CDKA activity in PES-, BOP1-, and WDR12-deficient seedlings (Fig. 6A, B).

### Real-time quantitative RT-PCR analyses of cell cycle-related gene expression

The E2F-RBR pathway plays a critical role in cell-cycle progression by regulating the G_1_/S transition in plants (Inzé and De Veylder, 2006; De Veylder *et al.*, 2007; Sablowski and Dornelas, 2014). RBR interacts with D cyclins (CycD) through a conserved LxCxE motif, and is phosphorylated by the CDKA/CycD complex depending on the cell-cycle phase (Boniotti and Gutierrez, 2001; Nakagami *et al.*, 2002). In a hypophosphorylated form, RBR interacts with the E2F/DP complex to repress transcription of the E2F target genes required for S phase entry, DNA replication, cell-cycle progression, and chromatin dynamics (Egelkrout *et al.*, 2002; Ramirez-Parra *et al.*, 2003; Kuwabara and Gruissem, 2014). Plant E2Fs are activated following RBR phosphorylation and function as transcriptional regulators; E2Fa and E2Fb are transcriptional activators and positive regulators of the cell cycle, whereas E2Fc is a transcriptional repressor and suppressor of cell division (Inzé and De Veylder, 2006; De Veylder *et al.*, 2007).

**Fig. 7.**
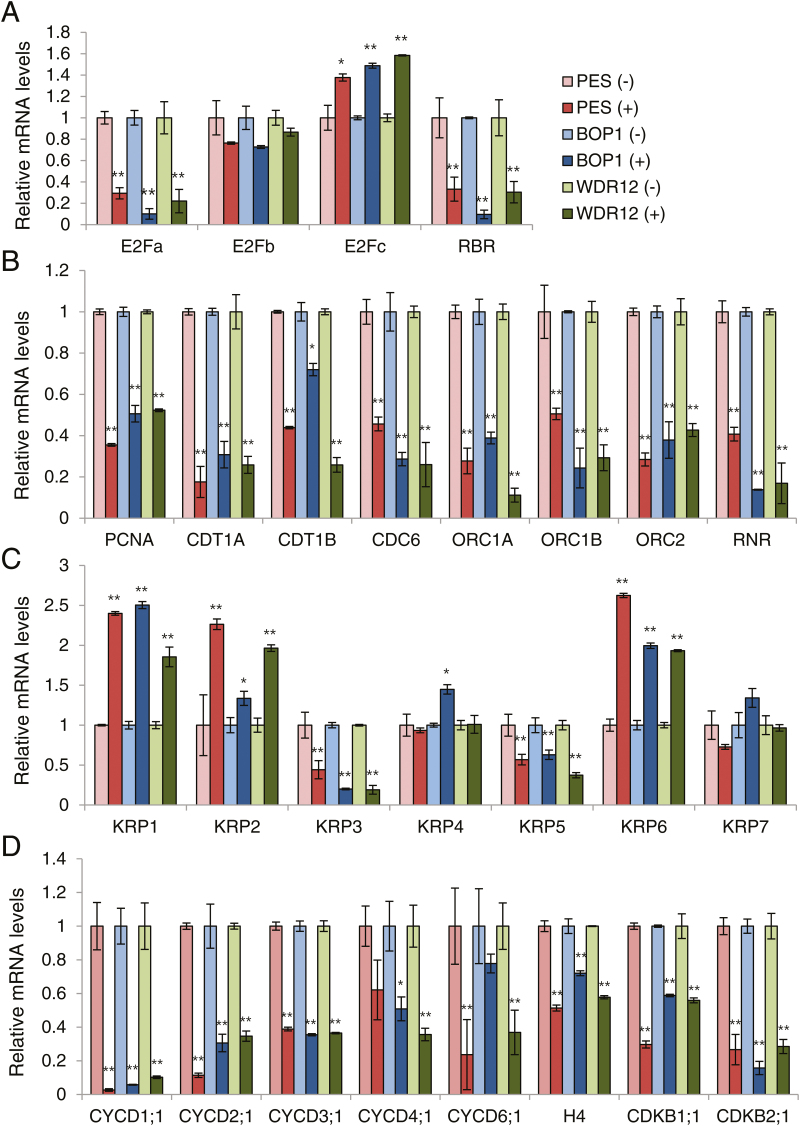
Expression of cell cycle-related genes. Real-time quantitative RT-PCR analyses were performed using the first leaves of the *PES* (#28), *BOP1* (#7), and *WDR12* (#8) RNAi seedlings sprayed with ethanol (–) or 20 µM DEX (+). Transcript levels are quantified relative to (–)DEX samples using *UBC10* mRNA levels as a control. Data points represent means ±SD of three experiments. Asterisks denote statistical significance of the differences between the (–)DEX and (+)DEX samples: *, *P*≤0.05; **, *P*≤0.01. (A) E2F/RBR pathway genes. (B) S-phase genes. (C) KRP family genes. (D) CycD family, histone H4, and CDKB genes. (This figure is available in colour at *JXB* online.)

We examined cell cycle-related gene expression in the RNAi plants (Fig. 7). The *PES* (#28), *BOP1* (#7), and *WDR12* (#8) RNAi seedlings at 3 DAC in soil were sprayed with ethanol or 20 µM DEX for 5 d, and the first true leaves were harvested for real-time quantitative RT-PCR. Among the E2F-RBR pathway genes, transcript levels of *E2Fa*, *E2Fb*, and *RBR* were reduced, whereas *E2Fc* transcript levels were elevated in all RNAi lines following DEX treatment (Fig. 7A). Transcript levels of S phase-specific genes, including proliferating cell nuclear antigen (*PCNA*), chromatin licensing and DNA replication factor 1A (*CDT1A*), *CDT1B*, cell division cycle 6 (*CDC6*), origin recognition complex 1A (*ORC1A*), *ORC1B*, *ORC2*, and ribonucleotide reductase (*RNR*) were all significantly reduced in PES-, BOP1-, and WDR12-depleted leaves, suggesting reduced cell proliferation (Fig. 7B). Among seven Arabidopsis genes encoding Kip-related proteins (KRPs), which inhibit the activity of CDK/CycD complexes, *KRP1*, *KRP2*, and *KRP6* were significantly up-regulated in DEX-treated samples, whereas expression of other *KRP* genes either decreased or remained constant (Fig. 7C). Furthermore, DEX treatment led to down-regulation of all of the CycD family genes tested (Fig. 7D). Transcript levels of histone H4, CDKB1;1, and CDKB2;1, all of which are expressed in actively dividing cells (Boudolf *et al.*, 2004), were also reduced in the first leaves of the RNAi plants following DEX treatment (Fig. 7D). It is known that CDKA and the E2F-RBR pathway play important roles in cell-cycle progression from G_1_ to S phase in plant cells (Inzé and De Veylder, 2006; De Veylder *et al.*, 2007; Sablowski and Dornelas, 2014). Reduced CDKA activity (Fig. 6) and the observed gene expression profiles suggest that impaired ribosome biogenesis caused by PeBoW deficiency inhibits the G_1_/S phase transition.

### Expression of cell cycle-related genes during the early response to PeBoW silencing

To examine the early effects of *PeBoW* silencing on expression of cell-cycle genes, an Arabidopsis liquid culture system was employed (see Supplementary Fig. S5A). *PES* (#28), *BOP1* (#7), and *WDR12* (#8) RNAi seedlings were grown in liquid culture, and at 7 d after sowing (DAS), the seedlings were treated with ethanol (–DEX) or 20 µM DEX for 12h. Real-time quantitative RT-PCR suggested that 12-h DEX treatment caused significant silencing of their corresponding genes in the first leaves (Supplementary Fig. S5B). Silencing of the *PeBoW* genes subsequently caused visible changes in gene expression patterns of S-phase genes and cell cycle-related genes in the first leaves (Supplementary Fig. S6). Expression of *E2Fa* and all of the S phase-specific genes tested (*PCNA*, *CDT1A*, *CDT1B*, *CDC6*, *ORC1A*, *ORC1B*, *ORC2*, and *RNR*) was significantly reduced, while expression of *KRP1* was up-regulated. Furthermore, histone *H4*, *CDKB1;1*, and *CDKB2;1*, as well as most of the *CycD* family genes tested except *CYCD3;1*, were down-regulated upon 12-h DEX treatment (Supplementary Fig. S6). Gene expression profiles after 24-h DEX treatment mostly mimicked the patterns observed after 12-h treatment, but the differences between (–)DEX and (+)DEX samples were more pronounced (Supplementary Fig. S7). Noticeably, 24-h DEX treatment caused down-regulation of *E2Fb* and *E2Fc*, and up-regulation of *KRP7*. *CYCD3;1* transcript levels, which were up-regulated after 12-h DEX treatment, changed to the control levels after 24-h treatment. Thus, *PeBoW* silencing caused rapid transcriptional modification of the cell-cycle genes in the first leaves, the patterns of which suggest that nucleolar stress immediately inhibits cell cycle progression. Furthermore, several key cell-cycle modulators, such as *E2Fb*, *E2Fc*, and *KRPs*, exhibited different transcriptional modulation depending on the duration of DEX treatment and plant growth/culture conditions (Fig. 7, Supplementary Figs S6 and S7).

We next examined the early effect of *PeBoW* silencing on cell proliferation by analyzing the first leaves of *PES* (#28), *BOP1* (#7), and *WDR12* (#8) RNAi seedlings (7 DAS) grown in liquid culture, after treatment with ethanol (–DEX) or 20 µM DEX for 24h (see Supplementary Fig. S8). The average leaf area, abaxial epidermal cell area, and the estimated epidermal cell number all increased within the 24-h period in all of the ethanol-treated RNAi lines (–DEX). However, those values barely changed in DEX-treated samples, suggesting blocked cell division and expansion. Collectively, these results suggest that the nucleolar stress caused by PeBoW depletion induces rapid transcriptional changes of cell-cycle genes, leading to almost immediate arrest of cell proliferation and expansion in actively dividing leaf cells.

### Expression of auxin- and jasmonic acid-related genes during the early response to *PeBoW* silencing

**Fig. 8.**
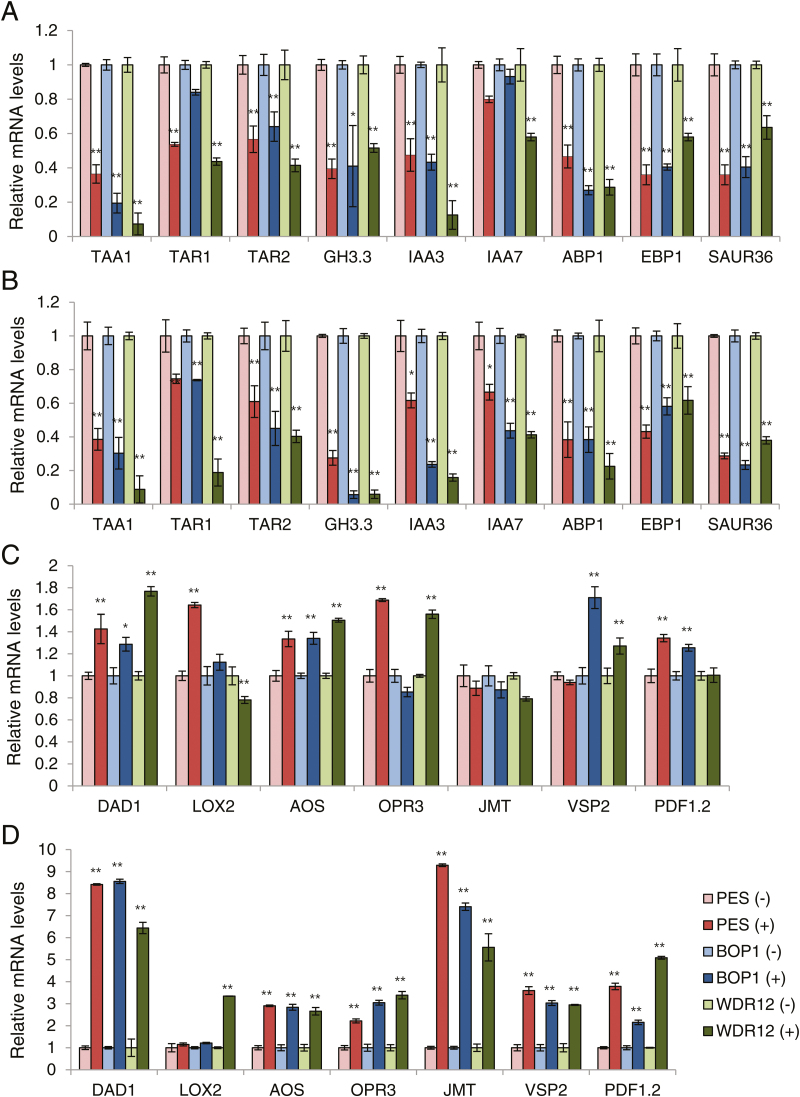
Real-time quantitative RT-PCR analyses to determine transcript levels of auxin- and JA-related genes after 12- and 24-h DEX treatments. The *PES* (#28), *BOP1* (#7), and *WDR12* (#8) RNAi seedlings (7 DAS) grown in liquid culture were treated with ethanol (–) or 20 µM DEX (+) for 12h or 24h. The first leaves were collected for the analyses. Transcript levels were quantified relative to (–)DEX samples using *UBC10* mRNA levels as a control. Each value represents the mean ±SD of three replicates per experiment. *, *P*≤0.05; **, *P*≤0.01. (A) Auxin-related genes after 12-h DEX treatment. (B) Auxin-related genes after 24-h DEX treatment. (C) JA-related genes after 12-h DEX treatment. (D) JA-related genes after 24-h DEX treatment. (This figure is available in colour at *JXB* online.)

Since auxin is a major positive regulator of cell division and cell expansion in plants, we next tested whether transcriptional changes of auxin-related genes are involved in the early response to *PeBoW* silencing. *PES* (#28), *BOP1* (#7), and *WDR12* (#8) RNAi seedlings (7 DAS) grown in liquid culture were treated with ethanol (–DEX) or 20 µM DEX for 12h or 24h, and real-time quantitative RT-PCR was performed with RNA isolated from the first leaves. 12-h DEX treatment caused a visible reduction in transcript levels of auxin biosynthesis-related genes, *TAA1* (tryptophan aminotransferase of Arabidopsis 1), *TAR1* (tryptophan aminotransferase related 1) and *TAR2*, and those of auxin-responsive genes, *GH3.3*, *IAA3* (indole-3-acetic acid protein 3), *IAA7*, *EBP1* (ErbB3 binding protein 1), and *SAUR36* (small auxin-up RNA 36) in all of the RNAi lines (Fig. 8A). Expression of *ABP1* encoding a putative auxin receptor was also down-regulated after 12-h DEX treatment. 24-h DEX treatment mostly caused a similar reduction in transcript levels compared to 12-h treatment, while several genes, such as *GH3.3* and *IAA7*, were further down-regulated (Fig. 8B). Thus, leaf cells’ early response to nucleolar stress includes rapid inhibition of auxin biosynthesis and signaling, which would negatively affect cell growth and proliferation.

Previous studies have suggested that the stress hormone jasmonic acid (JA) inhibits plant cell-cycle progression, repressing both cell division and expansion (Świa̧tek *et al.*, 2002, 2004; Zhang and Turner, 2008; Noir *et al.*, 2013; Wasternack and Hause, 2013). We tested if the nucleolar stress caused by PeBoW deficiency activates transcriptional changes of JA-related genes in the first leaves. 24-h DEX treatment resulted in significant transcriptional up-regulation of JA biosynthesis-related genes, *DAD1* (defective in anther dehiscence 1), *LOX2* (lipoxygenase 2), *AOS* (allene oxide synthase), *OPR3* (12-oxo-phytodienoic acid reductase 3) and *JMT* (*S*-adenosyl-L-methionine:jasmonic acid carboxyl methyltransferase), and JA-responsive genes, *VSP2* (vegetative storage protein 2) and *PDF1.2* (plant defensin 1.2), in all of the RNAi lines (Fig. 8D). DEX treatment for 12h caused only a mild up-regulation of most JA-related genes tested (Fig. 8C). Furthermore, the RNAi seedlings treated with 5 µM DEX for 6 d contained elevated endogenous JA contents, ~4.9 to ~15-fold higher than those of (–)DEX seedlings, consistent with the transcriptional up-regulation described above (see Supplementary Fig. S9). Cellular contents of another stress hormone abscisic acid were not significantly changed. Thus, nucleolar stress caused by PeBoW deficiency upregulated JA biosynthesis and signaling. Collectively, these results suggest an involvement of phytohormones, such as auxin and JA, in suppression of cell division and cell expansion in early stages of the nucleolar stress response in plants.

## Discussion

In this study, we characterized further the protein structures and *in planta* functions of the nucleolar proteins PES, BOP1, and WDR12, and examined the effects of their deficiency on cell growth and proliferation. We identified protein domains of BOP1 and WDR12 that are required for nucleolar localization, protein interactions, and ribosome co-fractionation. Depletion of the PeBoW components involved in ribosome biogenesis caused reduced CDKA activity with hypophosphorylation of RBR, and rapid changes in gene expression profiles of cell cycle genes and auxin- and jasmonic acid-related genes, leading to immediate inhibition of leaf cell growth and proliferation.

Cell growth and cell proliferation are tightly linked, as cells cannot divide without reaching a certain cell mass. The key determining factor in cell growth is ribosome biogenesis, which, not surprisingly, is closely linked to cell-cycle regulation. Disruption of PeBoW functions result in rapid cell-cycle arrest in mammals. Expression of BOP1∆, a dominant-negative form of BOP1, in human cells almost immediately blocks rRNA processing (Pestov *et al.*, 2001). The blocked 28S rRNA synthesis subsequently causes p53 activation, prohibiting cell-cycle progression through the G_1_/S checkpoint. In these cells, G_1_-specific CDK2 and CDK4 activities are down-regulated, the levels of CDK inhibitors p21^Cip1^ and p27^Kip1^ are elevated, and the cells lack hyperphosphorylated pRb (Pestov *et al.*, 2001). Similarly, down-regulation of PES in human breast cancer cells inhibits cell-cycle progression during the G_1_/S transition, dramatically reducing cyclin D1 and up-regulating the CDK inhibitor p27^Kip1^ (Li *et al.*, 2009). Conditional expression of a dominant negative mutant of WDR12 also induces rapid rRNA processing defects, followed by p53 activation and cell-cycle arrest (Hölzel *et al.*, 2005). In this study, PeBoW deficiencies in plants caused hypophosphorylation of RBR, down-regulation of *E2Fa* and *CycD* transcripts, and up-regulation of several *KRP* gene transcripts encoding plant CDK inhibitors in the first leaves, suggesting cell-cycle inhibition at the G_1_/S transition (Figs 6, 7). In particular, transcriptional changes of those G_1_/S phase regulators occurred rapidly, after only 12h of *PeBoW* silencing, followed by almost immediate suppression of cell proliferation, suggesting that plant cells possess a highly sensitive detection mechanism for anomalies in ribosome biogenesis (see Supplementary Figs S6, S8). Ribosome biogenesis is a complex process that is particularly sensitive to diverse cellular stresses such as disturbed metabolism and unfavorable/toxic environmental conditions (Antoniali *et al.*, 2014; Golomb *et al.*, 2014). The inhibitory effect of nucleolar stress on cell-cycle progression, mostly occurring at the G_1_/S checkpoint, may represent an inherent surveillance mechanism that prevents DNA synthesis under less favorable metabolic conditions.

Analyses of short-term and longer-term effects of *PeBoW* silencing on gene expression revealed common and differential modulation of the cell-cycle genes, probably influenced by the duration of stress, plant developmental stages, and plant culture conditions. Down-regulation of *E2Fa* was commonly observed after 12h, 24h, and 5 d of DEX treatment under different growth conditions, and thus appears to be one of the key regulatory events against plant nucleolar stress (Fig. 7A and Supplementary Figs S6A, S7A). In contrast, expression of *E2Fb* and *E2Fc* fluctuated depending on the conditions of growth. Transcriptional activators E2Fa and E2Fb co-ordinately control cell division and endoreduplication, but display specific gene expression patterns and have distinct roles during cell-cycle progression (Magyar *et al.*, 2005; Inzé and De Veylder, 2006; Sozzani *et al.*, 2006; De Veylder *et al.*, 2007; Sablowski and Dornelas, 2014). E2Fc functions as a repressor of cell division and inhibits expression of the S-phase genes, and plays a role in controlling the balance between cell proliferation and the switch to the endocycle program (del Pozo *et al.*, 2002, 2006). The observed fluctuation of *E2Fb* or *E2Fc* transcript levels following *PeBoW* silencing may represent dynamic temporal regulation of *E2Fb* or *E2Fc* transcription in response to progressive nucleolar stress. In a similar fashion, up-regulation of *CycD3;1* after 12-h DEX treatment may be an initial temporary response to *PeBoW* silencing (see Supplementary Fig. S6D), because *CycD3;1* and *E2Fa* expression are correlated, and are known to act in a common pathway in controlling the G_1_/S transition (de Jager *et al.*, 2009; Magyar *et al.*, 2012). Since *CYCD3;1* expression is induced by cytokinin (Riou-Khamlichi *et al.*, 1999), there is a possibility that *CycD3;1* was temporarily up-regulated in response to repressed auxin signaling in PeBoW-deficient plants.

We also observed that different *KRP* genes were up-regulated upon *PeBoW* silencing depending on the conditions; only *KRP1* was induced after 12h DEX treatment, *KRP1* and *KRP7* after 24h treatment, and *KRP1*, *KRP2*, and *KRP6* were induced after 5 d of DEX treatment (Fig. 7C and Supplementary Figs S6C, S7C). Thus, while different subsets of *KRP* genes responded to nucleolar stress under different conditions, *KRP1* was commonly up-regulated, suggesting its leading role under the stress. KRP1 can move between leaf cells, and misexpression of KRP1 blocks both G_1_/S transition and entry into mitosis in a cell context-dependent manner, and induces cell death (Schnittger *et al.*, 2003; Weinl *et al.*, 2005). Based on recent analyses of single-to-quintuple mutants of Arabidopsis *KRP* genes, gradual changes in phenotypes from single- to higher-order mutants suggest that plant KRPs mostly function redundantly in a dose-dependent manner (Cheng *et al.*, 2013). The finding that most of the E2F-regulated genes were up-regulated in Arabidopsis quintuple *KRP* mutants suggests that one mechanism of KRP function in cell division control is through regulation of E2Fs and E2F target genes (Cheng *et al.*, 2013). Collectively, the variable transcriptional modulation of the key cell-cycle regulators in PeBoW-deficient plants under different conditions suggests plasticity of the plant response mechanisms against nucleolar stress.

In this study, nucleolar stress caused by PeBoW depletion induced rapid changes of gene expression in the first leaves that suggest down-regulation of auxin biosynthesis and signaling, and up-regulation of JA biosynthesis and signaling (Fig. 8). Simultaneously, epidermal cell proliferation in the first leaves was blocked (see Supplementary Fig. S8). Therefore, the opposite modulation of these phytohormone signals may act as an antimitogenic signal, inhibiting cell-cycle progression and causing rapid repression of cell proliferation in PeBoW-deficient plants. Auxin is a major regulator of plant growth and development, controlling both cell division and cell expansion. Auxin induces the expression of the core cell-cycle regulators, such as *CycD*, *CDKA*, and *E2Fa*, and reduces the expression of several *KRP* genes during shoot and root development, regulating the G_1_/S transition (Himanen *et al.*, 2002; Braun *et al.*, 2008; Perrot-Rechenmann, 2010). Auxin also increases E2Fb protein stability by modulating its proteolysis (Magyar *et al.*, 2005). Auxin signaling pathways controlling the cell cycle may involve the auxin binding protein ABP1 and the AUX/IAA/SCF^TIR1AFB^ pathways, which control the G_1_/S transition by acting on the CycD/RBR/E2F pathway (David *et al.*, 2007; Tromas *et al.*, 2009; Perrot-Rechenmann, 2010). We observed that *ABP1* and several *AUX/IAA* genes were rapidly down-regulated upon *PeBoW* silencing, suggesting a possible involvement of the related pathways in cell-cycle repression during the early nucleolar stress response (Fig. 8A, B). Another phytohormone that seems to be associated with PeBoW deficiency is jasmonic acid (JA), based on the stimulated JA-related gene expression and the elevated JA contents (Fig. 8C, D and Supplementary Fig. S9). JA inhibits cell-cycle progression in synchronized tobacco BY-2 cells by blocking both G_1_/S and G_2_/M transitions (Świa̧tek *et al.*, 2002). Methyl jasmonate suppresses Arabidopsis leaf growth by inhibiting cell proliferation and expansion, arresting leaf cells in the G_1_ phase (Noir *et al.*, 2013). Treatment with coronatine, a high-affinity agonist of the JA receptor, also rapidly arrests Arabidopsis leaf growth, repressing the genes controlling cell division and expansion, such as D-type cyclins involved in the G_1_/S transition (Attaran *et al.*, 2014). However, JA signaling components directly linked to the cell-cycle machinery are still unclear. Although not explored in this study, other phytohormones may also play a role in linking nucleolar status to cell-cycle control, either directly or indirectly through hormone cross-talk. Further studies would elucidate the signaling cascades linking nucleolar stress to the cell-cycle in plants.

## Supplementary Data

Supplementary data are available at *JXB* online

Table S1. Primers used in this study.

Table S2. Data points for the kinematic analyses shown in Fig. 5.

Figure S1. Growth arrest phenotypes of *PES*-silenced plants.

Figure S2. Characterization of DEX-inducible *PES* (#38), *BOP1* (#10), and *WDR12* (#10) RNAi lines.

Figure S3. EtBr staining of total rRNA.

Figure S4. Unedited full images of Fig. 3A, B.

Figure S5. Gene silencing in RNAi seedlings grown in liquid culture.

Figure S6. Real-time quantitative RT-PCR analyses for the expression of cell cycle-related genes after 12-h DEX treatment.

Figure S7. Real-time quantitative RT-PCR analyses for the expression of cell cycle-related genes after 24-h DEX treatment.

Figure S8. Leaf cell division and expansion after 24-h DEX treatment.

Figure S9. Endogenous JA contents of the RNAi seedlings.

Supplementary Data

## References

[CIT0001] Adams CC Jakovljevic J Roman J Harnpicharnchai P Woolford JL Jr 2002 *Saccharomyces cerevisiae* nucleolar protein Nop7p is necessary for biogenesis of 60S ribosomal subunits. RNA 8, 150–165. 11911362 10.1017/s1355838202010026 PMC1370239

[CIT0002] Ahn CS Ahn H-K Pai H-S 2015 Overexpression of the PP2A regulatory subunit Tap46 leads to enhanced plant growth through stimulation of the TOR signaling pathway. Journal of Experimental Botany 66, 827–840. 25399018 10.1093/jxb/eru438 PMC4321543

[CIT0003] Ahn CS Han JA Lee H-S Lee S Pai H-S 2011 The PP2A regulatory subunit Tap46, a component of the TOR signaling pathway, modulates growth and metabolism in plants. The Plant Cell 23, 185–209. 21216945 10.1105/tpc.110.074005 PMC3051261

[CIT0004] Allende ML Amsterdam A Becker T Kawakami K Gaiano N Hopkins N 1996 Insertional mutagenesis in zebrafish identifies two novel genes, pescadillo and dead eye, essential for embryonic development. Genes & Development 10, 3141–3155. 8985183 10.1101/gad.10.24.3141

[CIT0005] Anastasiou E Lenhard M 2007 Growing up to one’s standard. Current Opinion in Plant Biology 10, 63–69. 17134936 10.1016/j.pbi.2006.11.002

[CIT0006] Antoniali G Lirussi L Poletto M Tell G 2014 Emerging roles of the nucleolus in regulating the DNA damage response: the noncanonical DNA repair enzyme APE1/Ref-1 as a paradigmatical example. Antioxidants & Redox Signaling 20, 621–639. 23879289 10.1089/ars.2013.5491 PMC3901381

[CIT0007] Aoyama T Chua N-H 1997 A glucocorticoid-mediated transcriptional induction system in transgenic plants. The Plant Journal 11, 605–612. 9107046 10.1046/j.1365-313x.1997.11030605.x

[CIT0008] Attaran E Major IT Cruz JA Rosa BA Koo AJ Chen J Kramer DM He SY Howe GA 2014 Temporal dynamics of growth and photosynthesis suppression in response to jasmonate signaling. Plant Physiology 165, 1302–1314. 24820026 10.1104/pp.114.239004 PMC4081338

[CIT0009] Boisvert FM van Koningsbruggen S Navascués J Lamond AI 2007 The multifunctional nucleolus. Nature Reviews Molecular Cell Biology 8, 574–585. 17519961 10.1038/nrm2184

[CIT0010] Boniotti MB Gutierrez C 2001 A cell-cycle-regulated kinase activity phosphorylates plant retinoblastoma protein and contains, in *Arabidopsis*, a CDKA/cyclin D complex. The Plant Journal 28, 341–350. 11722776 10.1046/j.1365-313x.2001.01160.x

[CIT0011] Boudolf V Vlieghe K Beemster GT Magyar Z Torres Acosta JA Maes S Van Der Schueren E Inzé D De Veylder L 2004 The plant-specific cyclin-dependent kinase CDKB1;1 and transcription factor E2Fa-DPa control the balance of mitotically dividing and endoreduplicating cells in *Arabidopsis*. The Plant Cell, 16, 2683–2692. 15377755 10.1105/tpc.104.024398 PMC520964

[CIT0012] Braun N Wyrzykowska J Muller P David K Couch D Perrot-Rechenmann C Fleming AJ 2008 Conditional repression of AUXIN BINDING PROTEIN1 reveals that it coordinates cell division and cell expansion during postembryonic shoot development in Arabidopsis and tobacco. The Plant Cell 20, 2746–2762. 18952781 10.1105/tpc.108.059048 PMC2590743

[CIT0013] Brown JW Shaw PJ 2008 The role of the plant nucleolus in pre-mRNA processing. Current Topics in Microbiology and Immunology 326, 291–311. 18630759 10.1007/978-3-540-76776-3_16 PMC7121088

[CIT0014] Cheng Y Cao L Wang S 2013 Downregulation of multiple CDK inhibitor ICK/KRP genes upregulates the E2F pathway and increases cell proliferation, and organ and seed sizes in *Arabidopsis*. The Plant Journal 75, 642–655. 23647236 10.1111/tpj.12228

[CIT0015] Cho HK Ahn CS Lee HS Kim JK Pai HS 2013 Pescadillo plays an essential role in plant cell growth and survival by modulating ribosome biogenesis. The Plant Journal 76, 393–405. 23909681 10.1111/tpj.12302

[CIT0016] Chung KY Cheng IK Ching AK Chu JH Lai PB Wong N 2011 Block of proliferation 1 (BOP1) plays an oncogenic role in hepatocellular carcinoma by promoting epithelial-to-mesenchymal transition. Hepatology 54, 307–318. 21520196 10.1002/hep.24372

[CIT0017] David KM Couch D Braun N Brown S Grosclaude J Perrot-Rechenmann C 2007 The auxin-binding protein 1 is essential for the control of cell cycle. The Plant Journal 50, 197–206. 17376160 10.1111/j.1365-313X.2007.03038.x

[CIT0018] de Jager SM Scofield S Huntley RP Robinson AS den Boer BG Murray JA 2009 Dissecting regulatory pathways of G1/S control in *Arabidopsis*: common and distinct targets of CYCD3;1, E2Fa and E2Fc. Plant Molecular Biology 71, 345–365. 19662336 10.1007/s11103-009-9527-5

[CIT0019] De Veylder L Beeckman T Beemster GTS 2001 Functional analysis of cyclin-dependent kinase inhibitors of *Arabidopsis*. The Plant Cell 13, 1653–1668. 11449057 10.1105/TPC.010087 PMC139548

[CIT0020] De Veylder L Beeckman T Inzé D 2007 The ins and outs of the plant cell cycle. Nature Reviews Molecular Cell Biology 8, 655–665. 17643126 10.1038/nrm2227

[CIT0021] del Pozo JC Boniotti MB Gutierrez C 2002 *Arabidopsis* E2Fc functions in cell division and is degraded by the ubiquitin-SCF(SKP2) pathway in response to light. The Plant Cell 14, 3057–3071. 12468727 10.1105/tpc.006791 PMC151202

[CIT0022] del Pozo JC Diaz-Trivino S Cisneros N Gutierrez C 2006 The balance between cell division and endoreplicationdepends on E2Fc-DPB, transcription factors regulated by the ubiquitin-SCF^SKP2A^ pathway in *Arabidopsis*. The Plant Cell 18, 2224–2235. 16920782 10.1105/tpc.105.039651 PMC1560920

[CIT0023] Donati G Montanaro L Derenzini M 2012 Ribosome biogenesis and control of cell proliferation: p53 is not alone. Cancer Research 72, 1602–1607. 22282659 10.1158/0008-5472.CAN-11-3992

[CIT0024] Ebersberger I Simm S Leisegang MS Schmitzberger P Mirus O von Haeseler A Bohnsack MT Schleiff E 2014 The evolution of the ribosome biogenesis pathway from a yeast perspective. Nucleic Acids Research 42, 1509–1523. 24234440 10.1093/nar/gkt1137 PMC3919561

[CIT0025] Egelkrout EM Mariconti L Settlage SB Cella R Robertson D Hanley-Bowdoin L 2002 Two E2F elements regulate the proliferating cell nuclear antigen promoter differently during leaf development. The Plant Cell 14, 3225–3236. 12468739 10.1105/tpc.006403 PMC151214

[CIT0026] Golomb L Volarevic S Oren M 2014 p53 and ribosome biogenesis stress: the essentials. FEBS Letters 588, 2571–2579. 24747423 10.1016/j.febslet.2014.04.014

[CIT0027] Grimm T Hölzel M Rohrmoser M Harasim T Malamoussi A Gruber-Eber A Kremmer E Eick D 2006 Dominant-negative Pes1 mutants inhibit ribosomal RNA processing and cell proliferation via incorporation into the PeBoW-complex. Nucleic Acids Research 34, 3030–3043. 16738141 10.1093/nar/gkl378 PMC1474060

[CIT0028] Henras AK Soudet J Gérus M Lebaron S Caizergues-Ferrer M Mougin A Henry Y 2008 The post-transcriptional steps of eukaryotic ribosome biogenesis. Cellular and Molecular Life Sciences 65, 2334–2359. 18408888 10.1007/s00018-008-8027-0 PMC11131730

[CIT0029] Himanen K Boucheron E Vanneste S de Almeida Engler J Inzé D Beeckman T 2002 Auxin-mediated cell cycle activation during early lateral root initiation. The Plant Cell 14, 2339–2351. 12368490 10.1105/tpc.004960 PMC151221

[CIT0030] Hölzel M Rohrmoser M Schlee M 2005 Mammalian WDR12 is a novel member of the Pes1-BOP1 complex and is required for ribosome biogenesis and cell proliferation. Journal of Cell Biology 170, 367–378. 16043514 10.1083/jcb.200501141 PMC2171466

[CIT0031] Horiguchi G Ferjani A Fujikura U Tsukaya H 2006 Coordination of cell proliferation and cell expansion in the control of leaf size in *Arabidopsis thaliana*. Journal of Plant Research 119, 37–42. 16284709 10.1007/s10265-005-0232-4

[CIT0032] Horiguchi G Van Lijsebettens M Candela H Micol JL Tsukaya H 2012 Ribosomes and translation in plant developmental control. Plant Science 191–192, 24–34. 10.1016/j.plantsci.2012.04.008 22682562

[CIT0033] Huang M Ji Y Itahana K Zhang Y Mitchell B 2008 Guanine nucleotide depletion inhibits pre-ribosomal RNA synthesis and causes nucleolar disruption. Leukemia Research 32, 131–141. 17462731 10.1016/j.leukres.2007.03.025 PMC4552191

[CIT0034] Inzé D De Veylder L 2006 Cell cycle regulation in plant development. Annual Review of Genetics 40, 77–105. 10.1146/annurev.genet.40.110405.090431 17094738

[CIT0035] James A Wang Y Raje H Rosby R DiMario P 2014 Nucleolar stress with and without p53. Nucleus 5, 402–26. 25482194 10.4161/nucl.32235 PMC4164484

[CIT0036] Jeon Y Park YJ Cho HK Jung HJ Ahn TK Kang H Pai H-S 2015 The nucleolar GTPase nucleostemin-like 1 plays a role in plant growth and senescence by modulating ribosome biogenesis. Journal of Experimental Botany 66, 6297–6310. 26163696 10.1093/jxb/erv337 PMC4588883

[CIT0037] Karbstein K 2011 Inside the 40S ribosome assembly machinery. Current Opinion in Chemical Biology 15, 657–663. 21862385 10.1016/j.cbpa.2011.07.023 PMC3329787

[CIT0038] Killian A Le Meur N Sesboüé R Bourguignon J Bougeard G Gautherot J Bastard C Frébourg T Flaman JM 2004 Inactivation of the RRB1-Pescadillo pathway involved in ribosome biogenesis induces chromosomal instability. Oncogene 23, 8597–8602. 15467761 10.1038/sj.onc.1207845

[CIT0039] Kressler D Hurt E Bassler J 2010 Driving ribosome assembly. Biochimica et Biophysica Acta 1803, 673–683. 19879902 10.1016/j.bbamcr.2009.10.009

[CIT0040] Kuwabara A Gruissem W 2014 *Arabidopsis* RETINOBLASTOMA-RELATED and Polycomb group proteins: cooperation during plant cell differentiation and development. Journal of Experimental Botany 65, 2667–2676. 24638900 10.1093/jxb/eru069

[CIT0041] Lam YW Trinkle-Mulcahy L 2015 New insights into nucleolar structure and function. F1000Prime Reports 7, 48. 26097721 10.12703/P7-48 PMC4447046

[CIT0042] Lapik YR Fernandes CJ Lau LF Pestov DG 2004 Physical and functional interaction between Pes1 and BOP1 in mammalian ribosome biogenesis. Molecular Cell 15, 17–29. 15225545 10.1016/j.molcel.2004.05.020

[CIT0043] Lee H-J Park Y-J Seo PJ Kim J-H Sim H-J Kim S-G Park C-M 2015 Systemic immunity requires SnRK2.8-mediated nuclear import of NPR1 in *Arabidopsis*. The Plant Cell 27, 3425–3438. 26672073 10.1105/tpc.15.00371 PMC4707448

[CIT0044] Lerch-Gaggl A Haque J Li J Ning G Traktman P Duncan SA 2002 Pescadillo is essential for nucleolar assembly, ribosome biogenesis, and mammalian cell proliferation. Journal of Biological Chemistry 277, 45347–45355. 12237316 10.1074/jbc.M208338200

[CIT0045] Li J Yu L Zhang H Wu J Yuan J Li X Li M 2009 Down-regulation of pescadillo inhibits proliferation and tumorigenicity of breast cancer cells. Cancer Science 100, 2255–2260. 19764998 10.1111/j.1349-7006.2009.01325.x PMC11159139

[CIT0046] Lundin C North M Erixon K Walters K Jenssen D Goldman AS Helleday T 2005 Methyl methanesulfonate (MMS) produces heat-labile DNA damage but no detectable in vivo DNA double-strand breaks. Nucleic Acids Reserach 33, 3799–3811. 10.1093/nar/gki681 PMC1174933 16009812

[CIT0047] Magyar Z De Veylder L Atanassova A Bakó L Inzé D Bögre L 2005 The role of the *Arabidopsis* E2FB transcription factor in regulating auxin-dependent cell division. The Plant Cell 17, 2527–2541. 16055635 10.1105/tpc.105.033761 PMC1197432

[CIT0048] Magyar Z Horváth B Khan S Mohammed B Henriques R De Veylder L Bakó L Scheres B Bögre L 2012 *Arabidopsi*s E2FA stimulates proliferation and endocycle separately through RBR-bound and RBR-free complexes. The EMBO Journal 31, 1480–1493. 22307083 10.1038/emboj.2012.13 PMC3321179

[CIT0049] Mayer C Grummt I 2005 Cellular stress and nucleolar function. Cell Cycle 4, 1036–1038. 16205120 10.4161/cc.4.8.1925

[CIT0050] Miles TD Jakovljevic J Horsey EW Harnpicharnchai P Tang L Woolford JL Jr 2005 Ytm1, Nop7, and Erb1 form a complex necessary for maturation of yeast 66S preribosomes. Molecular and Cellular Biology 25, 10419–10432. 16287855 10.1128/MCB.25.23.10419-10432.2005 PMC1291219

[CIT0051] Missbach S Weis BL Martin R Simm S Bohnsack MT Schleiff E 2013 40S ribosome biogenesis co-factors are essential for gametophyte and embryo development. PLoS ONE 28, e54084. 23382868 10.1371/journal.pone.0054084 PMC3559688

[CIT0052] Nakagami H Kawamura K Sugisaka K Sekine M Shinmyo A 2002 Phosphorylation of retinoblastoma-related protein by the cyclin D/cyclin-dependent kinase complex is activated at the G1/S-phase transition in tobacco. The Plant Cell 14, 1847–1857. 12172026 10.1105/tpc.002550 PMC151469

[CIT0053] Noir S Bömer M Takahashi N Ishida T Tsui TL Balbi V Shanahan H Sugimoto K Devoto A 2013 Jasmonate controls leaf growth by repressing cell proliferation and the onset of endoreduplication while maintaining a potential stand-by mode. Plant Physiology 161, 1930–1951. 23439917 10.1104/pp.113.214908 PMC3613466

[CIT0054] Panse VG Johnson AW 2010 Maturation of eukaryotic ribosomes: acquisition of functionality. Trends in Biochemical Sciences 35, 260–266. 20137954 10.1016/j.tibs.2010.01.001 PMC2866757

[CIT0055] Perrot-Rechenmann C 2010 Cellular responses to auxin: division versus expansion. Cold Spring Harbor Perspectives in Biology 2, a001446. 20452959 10.1101/cshperspect.a001446 PMC2857164

[CIT0056] Pestov DG Stockelman MG Strezoska Z Lau LF 2001 ERB1, the yeast homolog of mammalian BOP1, is an essential gene required for maturation of the 25S and 5.8S ribosomal RNAs. Nucleic Acids Research 29, 3621–3630. 11522832 10.1093/nar/29.17.3621 PMC55883

[CIT0057] Ramirez-Parra E Fründt C Gutierrez C 2003 A genome-wide identification of E2F regulated genes in *Arabidopsis*. The Plant Journal 33, 801–811. 12609051 10.1046/j.1365-313x.2003.01662.x

[CIT0058] Revenkova E Masson J Koncz C Afsar K Jakovleva L Paszkowski J 1999 Involvement of *Arabidopsis thaliana* ribosomal protein S27 in mRNA degradation triggered by genotoxic stress. The EMBO Journal 18, 490–499. 9889204 10.1093/emboj/18.2.490 PMC1171142

[CIT0059] Riou-Khamlichi C Huntley R Jacqmard A Murray JA 1999 Cytokinin activation of Arabidopsis cell division through a D-type cyclin. Science 283, 1541–1544. 10066178 10.1126/science.283.5407.1541

[CIT0060] Rohrmoser M Hölzel M Grimm T Malamoussi A Harasim T Orban M Pfisterer I Gruber-Eber A Kremmer E Eick D 2007 Interdependence of Pes1, Bop1, and WDR12 controls nucleolar localization and assembly of the PeBoW complex required for maturation of the 60S ribosomal subunit. Molecular and Cellular Biology 27, 3682–3694. 17353269 10.1128/MCB.00172-07 PMC1899993

[CIT0061] Sablowski R Dornelas MC 2014 Interplay between cell growth and cell cycle in plants. Journal of Experimental Botany 65, 2703–2714. 24218325 10.1093/jxb/ert354

[CIT0062] Schnittger A Weinl C Bouyer D Schöbinger U Hülskamp M 2003 Misexpression of the cyclin-dependent kinase inhibitor ICK1/KRP1 in single-celled Arabidopsis trichomes reduces endoreduplication and cell size and induces cell death. The Plant Cell 15, 303–315. 12566574 10.1105/tpc.008342 PMC141203

[CIT0063] Shaw P Brown J 2012 Nucleoli: composition, function, and dynamics. Plant Physiology 158, 44–51. 22082506 10.1104/pp.111.188052 PMC3252080

[CIT0064] Sozzani R Maggio C Varotto S Canova S Bergounioux C Albani D Cella R 2006 Interplay between Arabidopsis activating factors E2Fb and E2Fa in cell cycle progression and development. Plant Physiology 140, 1355–1366. 16514015 10.1104/pp.106.077990 PMC1435807

[CIT0065] Strezoska Z Pestov DG Lau LF 2000 BOP1 is a mouse WD40 repeat nucleolar protein involved in 28S and 5. 8S RRNA processing and 60S ribosome biogenesis. Molecular and Cellular Biology 20, 5516–5528. 10891491 10.1128/mcb.20.15.5516-5528.2000 PMC86002

[CIT0066] Świa̧tek A Azmi A Stals H Inzé D Van Onckelen H 2004 Jasmonic acid prevents the accumulation of cyclin B1;1 and CDK-B in synchronized tobacco BY-2 cells. FEBS Letters 572, 118–122. 15304334 10.1016/j.febslet.2004.07.018

[CIT0067] Świa̧tek A Lenjou M Van Bockstaele D Inzé D Van Onckelen H 2002 Differential effect of jasmonic acid and abscisic acid on cell cycle progression in tobacco BY-2 cells. Plant Physiology 128, 201–211. 11788766 PMC148976

[CIT0068] Tromas A Braun N Muller P 2009 The AUXIN BINDING PROTEIN 1 is required for differential auxin responses mediating root growth. PLoS ONE 4, e6648. 19777056 10.1371/journal.pone.0006648 PMC2744284

[CIT0069] Tsai RY McKay RD 2005 A multistep, GTP-driven mechanism controlling the dynamic cycling of nucleostemin. Journal of Cell Biology 168, 179–184. 15657390 10.1083/jcb.200409053 PMC2171593

[CIT0070] Wasternack C Hause B 2013 Jasmonates: biosynthesis, perception, signal transduction and action in plant stress response, growth and development. An update to the 2007 review in Annals of Botany. Annals of Botany 111, 1021–1058. 23558912 10.1093/aob/mct067 PMC3662512

[CIT0071] Weinl C Marquardt S Kuijt SJ Nowack MK Jakoby MJ Hülskamp M Schnittger A 2005 Novel functions of plant cyclin-dependent kinase inhibitors, ICK1/KRP1, can act non-cell-autonomously and inhibit entry into mitosis. The Plant Cell 17, 1704–1722. 15749764 10.1105/tpc.104.030486 PMC1143071

[CIT0072] Weis BL Missbach S Marzi J Bohnsack MT Schleiff E 2014 The 60S associated ribosome biogenesis factor LSG1-2 is required for 40S maturation in *Arabidopsis thaliana*. The Plant Journal 80, 1043–1056. 25319368 10.1111/tpj.12703

[CIT0073] Woolford JL Baserga SJ 2013 Ribosome biogenesis in the yeast *Saccharomyces cerevisiae*. Genetics 195, 643–681. 24190922 10.1534/genetics.113.153197 PMC3813855

[CIT0074] Zhang Y Turner JG 2008 Wound-induced endogenous jasmonates stunt plant growth by inhibiting mitosis. PLoS ONE 3, e3699. 19002244 10.1371/journal.pone.0003699 PMC2577035

