# Supplementary material for: Functional characterization of the ribosome biogenesis factors PES, BOP1, and WDR12 (PeBoW), and mechanisms of defective cell growth and proliferation caused by PeBoW deficiency in Arabidopsis
Source: J Exp Bot. 2016 Jul 20;67(17):5217–32. doi: 10.1093/jxb/erw288 (PMC5014167; doi:10.1093/jxb/erw288)
Supplement: Supplementary Data [file supp_67_17_5217__index.html]

Functional characterization of the ribosome biogenesis factors PES, BOP1, and WDR12 (PeBoW), and mechanisms of defective cell growth and proliferation caused by PeBoW deficiency in Arabidopsis — Functional characterization of the ribosome biogenesis factors PES, BOP1, and WDR12 (PeBoW), and mechanisms of defective cell growth and proliferation caused by PeBoW deficiency in Arabidopsis — Supplementary Data 

# Functional characterization of the ribosome biogenesis factors PES, BOP1, and WDR12 (PeBoW), and mechanisms of defective cell growth and proliferation caused by PeBoW deficiency in Arabidopsis

## Supplementary Data

Data files

- supplementary\_tables\_S1\_S2\_figures\_S1\_S9.pdf - Supplementary Data
