# Supplementary material for: Functional characterization of the ribosome biogenesis factors PES, BOP1, and WDR12 (PeBoW), and mechanisms of defective cell growth and proliferation caused by PeBoW deficiency in Arabidopsis
Source: J Exp Bot. 2016 Jul 20;67(17):5217–32. doi: 10.1093/jxb/erw288 (PMC5014167; doi:10.1093/jxb/erw288)
Supplement: Supplementary Data [file supp_erw288_supplementary_tables_S1_S2_figures_S1_S9.pdf]

Supplementary Table S1. PCR primers used in this study

Primer names	Gene ID	Sequence (F)	Sequence (R)
UBC10	AT5G53300	atgggtccttcagagagtcct	tggaacaccttggtcctaaag
PES	AT5G14520	ccaggtcgcaagctcttaaa	catggtagtaagtgtgatggtttcc
BOP1	AT2G40360	tgccattggagattctaagagg	gttgtctcggatgaaacttgc
WDR12	AT5G15550	ttagttaattccaacgatgcagaa	caacagactcagctggatcaaa
PCNA	AT1G07370	tcatttgccctgaggtacatg	tactccacaaccactggcaac
CDT1A	AT2G31270	cttcatgttacgctcaatgcc	gcaagtctagcccgaaacact
CDT1B	AT3G54710	ttttcgcaaagctcatcctaa	cctttgaggagaattgaatggt
CDC6	AT2G29680	tccagtctctacctcaacacca	cctttttgctccctcgaaa
ORC1A	AT4G14700	agaagacgcgaatccagatg	atctgacaatcttcgatttctgg
ORC1B	AT4G12620	cactcctcaaaccccagaaa	cgagaaacatgacgagcaga
ORC2	AT2G37560	tgcccacaagctctctgata	tcaattgtagaagccgtttctct
RNR	AT3G23580	tccggaagcttttgttcaat	tgttaatccaggcatgagtcc
E2Fa	AT2G36010	cgttcatggttgtggtgttg	cgcctttctctttcgtgaag
E2Fb	AT5G22220	cgcttaagaacaggattcagtg	ctcagctgcgaggttttgta
E2Fc	AT1G47870	ggaaagcaggttggatgatct	ccgtcataaacatgtaccttctaca
RBR	AT3G12280	tgaactttacggcgcagact	cggaacccacgtttgtagtatt
KRP1	AT2G23430	cgtcttgtagtggaagcaatga	cgacgtttcagtgtcaccat
KRP2	AT3G50630	cgtggatttacgatgatttgaa	gcggcgagactctacatctt
KRP3	AT5G48820	tgaaatggaggagttctttgc	gggggatatcattcacaatgtc
KRP4	AT2G32710	ccctaggagtaatttggattcg	cactttcagaaacagaatcagacc
KRP5	AT3G24810	aaagtgagattgaagacttctttgc	ttgtccgagacaatgtcaaagt
KRP6	AT3G19150	gaaaccgaaaccgaaacctc	ccctcactcactggactcgt
KRP7	AT1G49620	gcaggcagagcttgatgact	tcattgacgatgtcgtagttgtact
CYCD1;1	AT1G70210	tcggtttctttacgctcgtc	aaggaacgagaatttcctcca
CYCD2;1	AT1G76540	caagcaatctttcagggagact	attgggtgtcccaaacaact
CYCD3;1	AT4g34160	cgcgtgtctctcattagctg	catactttgtctcctccacttgaa
CYCD4;1	AT5G65420	gccagcacaaccaaaggtat	cccattggg tgtttgtgaac
CYCD6;1	AT4G03270	ttggcctttttcattagtctcttt	tggtttaaactcgaggaaactga
H4	AT1G07820	gaaagggaggcaagggatta	cacgtaataccttacggtgtcg
CDKB1;1	AT3G54180	ttatacgcatgagattgttactctttg	ctccgaaccatctcagcaa
CDKB2;1	AT1G76540	cacagtggaaaccatcgactc	ggctcgtactgcagcatttta
DAD1	AT2G44810	aaacgtgcgcctatggttac	tcgacgctgtcatcttaacg
LOX2	AT3G45140	cttacccgcggatctcatc	actccatgttctgcggtctt
AOS	AT5G42650	caccggcgttagtcaaatct	ccggcggattctaagaaaa
OPR3	AT2G06050	gcgttgaacggagtaccaa	caggcacatgtgggaacc
JMT	AT1G19640	ggttcgttctacggacgttt	cctcacgacatggaacctg
VSP2	AT5G24770	cgtcgattcgaaaaccatct	ggcaccgtgtcgaagtctat
PDF1.2	AT5G44420	gagttgtgcgagaagccaagt	gttgcatgatccatgtttgg
TAA1	AT1G70560	agcagagctggagagcgttgtg	cttcatgttggcgagtctctcgag
TAR1	AT1G23320	cttcatgttggcgagtctctcgag	acgctggtcagagttatgagacacc
TAR2	AT4G24670	aaggttgtgtcagacagttgtggg	ggttgtggctcaaagaccctgc
GH3.3	AT2G23170	catcacagagttcctcacaagc	gtcggtccatgtcttcatca
IAA3	AT1G04240	aaaggctcagattgttggatggc	tgaccctcatgctcagattcattc
IAA7	AT1G04240	aagctaccaggatctttctgatgc	attccttgtgctccatagtttccc
ABP1	AT4G02980	ccaatctttgccaacagtacaa	ctcatgaccggtgtttttga
EBP1	AT3G51800	gtggtggaaagaagaagaaagc	gcattactacttgcgtccattg
SAUR36	AT2G45210	agccgaaaaagagtacggatt	gacccgttcgaaatctgaata

Supplementary Table S2

Supplementary Table S2. Data points for the kinematic analyses shown in Fig. 5.

A

	Average areas of first leaves (mm^2)					
	PES RNAi (#28)		BOP1 RNAi (#7)		WDR12 RNAi (#8)	
Stage	DEX (-)	DEX (+)	DEX (-)	DEX (+)	DEX (-)	DEX (+)
5 DAC	0.520±0.140	0.223±0.041	0.541±0.112	0.182±0.042	0.438±0.087	0.198±0.039
8 DAC	2.520±0.502	0.361±0.052	2.726±0.773	0.321±0.054	2.121±0.614	0.418±0.081
10 DAC	6.655±0.643	0.460±0.093	6.873±0.782	0.450±0.082	6.215±0.568	0.529±0.061
14 DAC	8.282±0.863	0.560±0.092	8.599±0.857	0.510±0.084	8.082±0.777	0.629±0.075

B

	Average areas of epidermal cells (μm^2)					
	PES RNAi (#28)		BOP1 RNAi (#7)		WDR12 RNAi (#8)	
Stage	DEX (-)	DEX (+)	DEX (-)	DEX (+)	DEX (-)	DEX (+)
5 DAC	609.683±126.6	344.42±151.8	687.171±135.6	281.098±94.8	605.929±127.7	287.57±93.2
8 DAC	1235.51±348.0	466.489±134.9	1420.861±329.0	420.270±137.6	1365.241±332.2	501.359±146.4
10 DAC	2396.211±593.2	547.61±314.6	2481.058±504.9	564.009±220.6	2314.344±607.0	602.855±221.6
14 DAC	2831.499±596.8	655.171±238.9	2909.276±553.1	618.309±215.1	2824.703±582.8	690.211±205.9

C

	Calculated numbers of epidermal cells per leaf					
	PES RNAi (#28)		BOP1 RNAi (#7)		WDR12 RNAi (#8)	
Stage	DEX (-)	DEX (+)	DEX (-)	DEX (+)	DEX (-)	DEX (+)
5 DAC	854.4	647.6	787.8	650.8	723.6	689.5
8 DAC	2040.3	774.0	1918.8	764.0	1554.2	835.3
10 DAC	2777.5	841.6	2770.5	799.2	2685.6	877.6
14 DAC	2925.0	856.2	2955.9	826.2	2861.2	911.5

Supplementary Figure S1

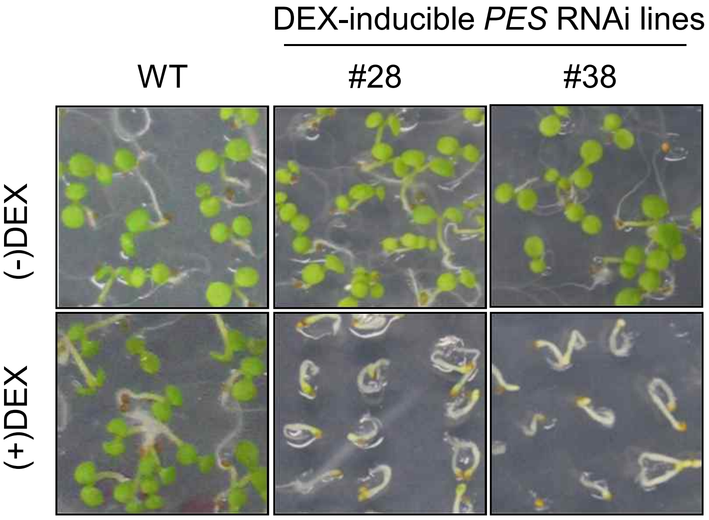

Supplementary Fig. S1. Growth arrest phenotypes of *PES*-silenced plants.

Growth defects of *Arabidopsis* dexamethasone (DEX)-inducible *PES* RNAi lines (#28 and #38). Seedlings were germinated on MS media that contained either ethanol (-DEX) or 10 µM DEX.

Supplementary Figure S2

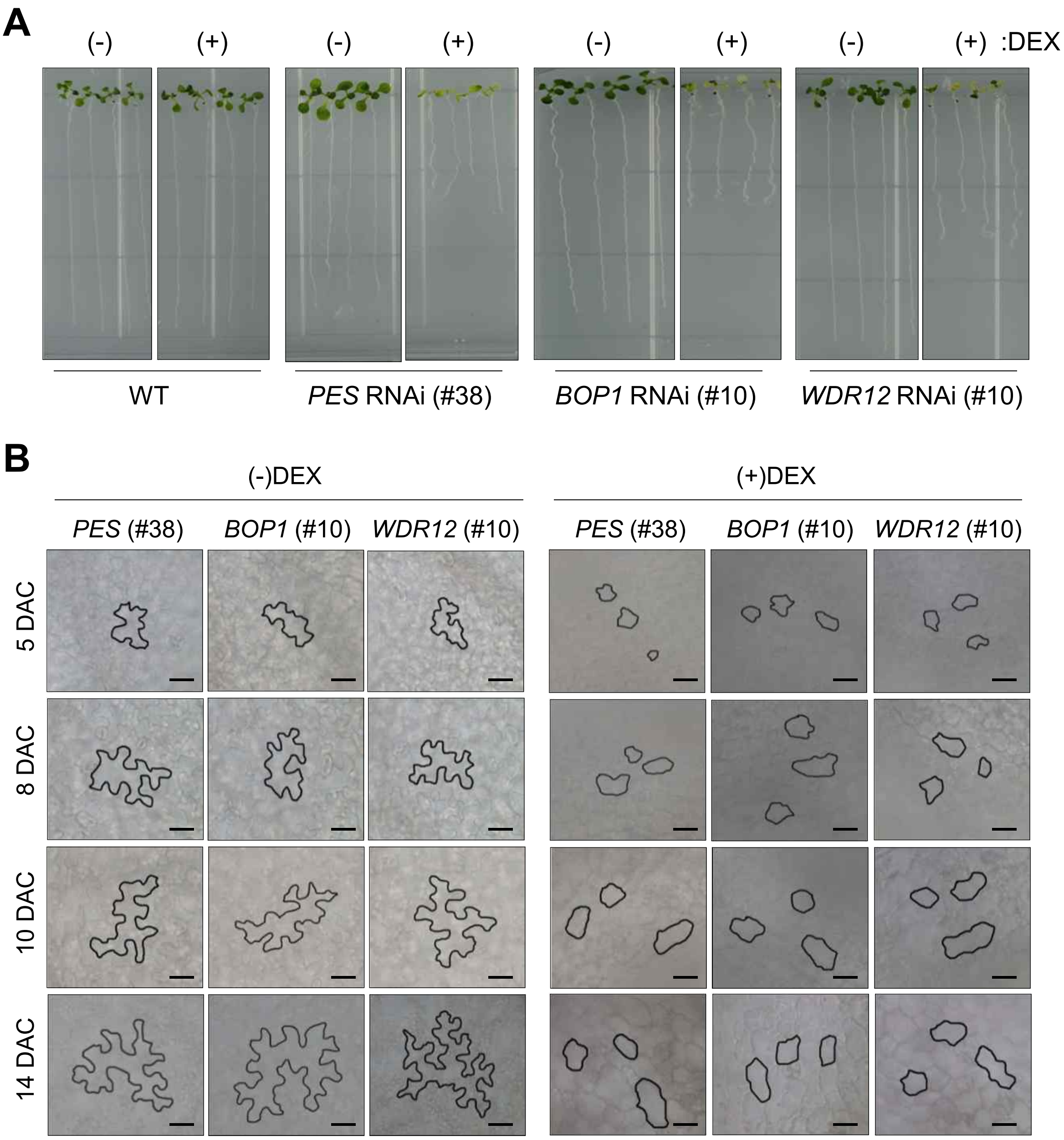

Supplementary Fig. S2. Characterization of DEX-inducible *PES* (#38), *BOP1* (#10), and *WDR12* (#10) RNAi lines.

(A) Retarded root growth and premature senescence of the RNAi seedlings upon DEX treatment. Seedlings were grown on MS media and then transferred to (-)DEX or (+)DEX media for vertical growth.

(B) Representative abaxial epidermal cells of the first leaves. The RNAi plants were grown in soil and sprayed with ethanol (-) or 20 μM DEX (+). The first leaves were collected from the plants at 5, 8, 10, and 14 days after cotyledon emergence (DAC). Individual cells are visualized by *black outlines* using ImageJ. Scale bars = 20 μm.

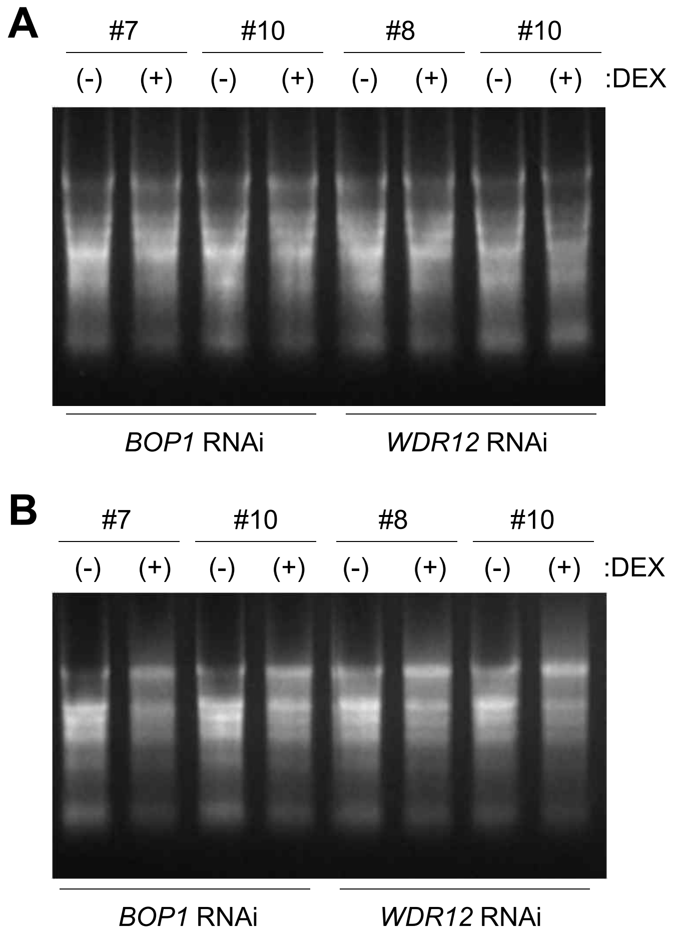

Supplementary Fig. S3. EtBr staining of total rRNA.

BOP1 (lines #7 and #10) and *WDR12* (lines #8 and #10) RNAi seedlings were grown on MS medium for 7 days and then transferred to MS medium containing either ethanol (-DEX) or 10 μM DEX. At 2 days (A) and 4 days after transfer (B), total RNA was isolated from equal fresh weights (50 mg) of seedlings, and separated by agarose gel electrophoresis. The agarose gel was stained with ethidium bromide (EtBr) to visualize total mature rRNAs.

Supplementary Figure S4

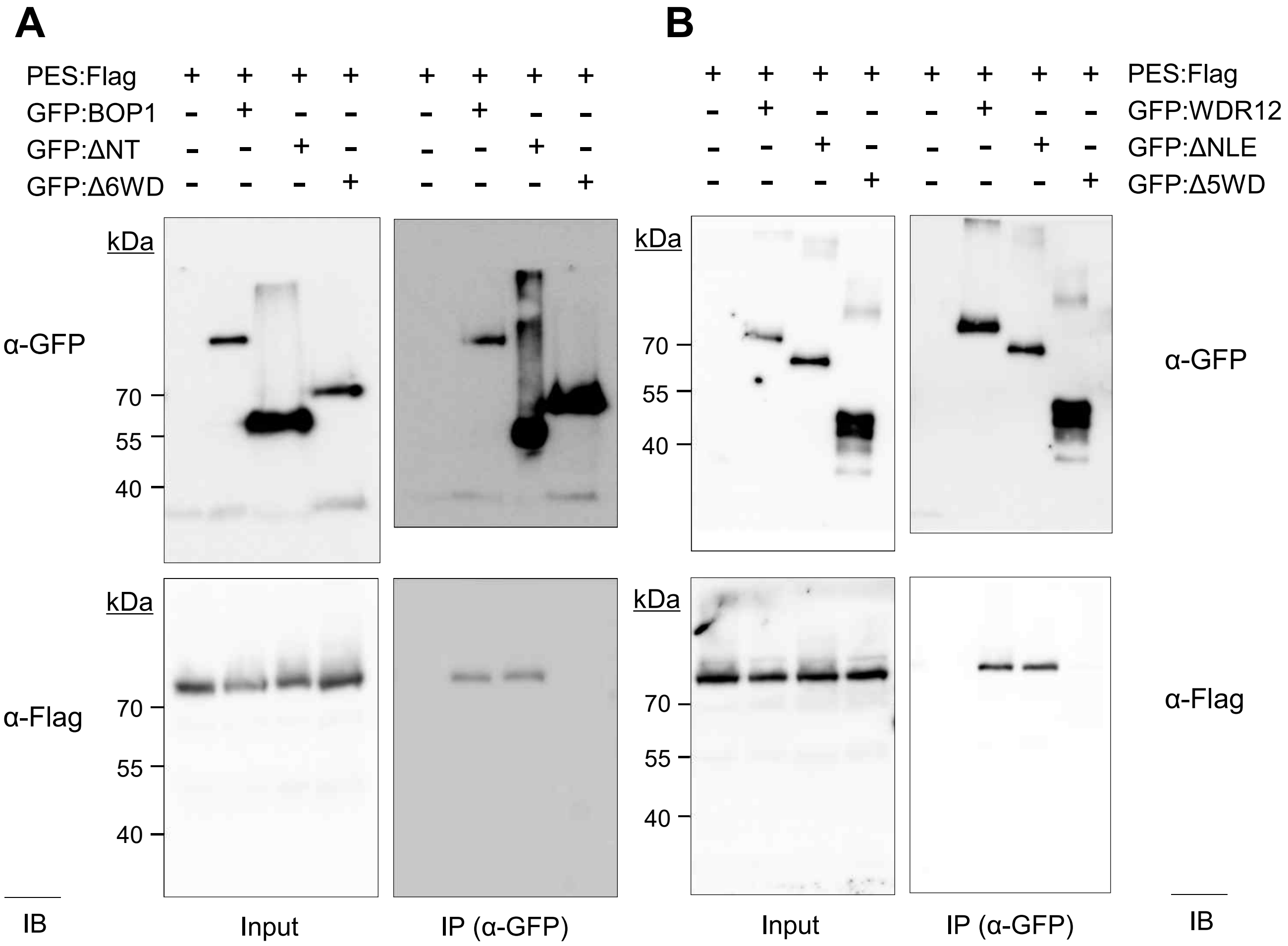

Supplementary Fig. S4. Unedited full images of Figure 3A and B.

Supplementary Figure S5

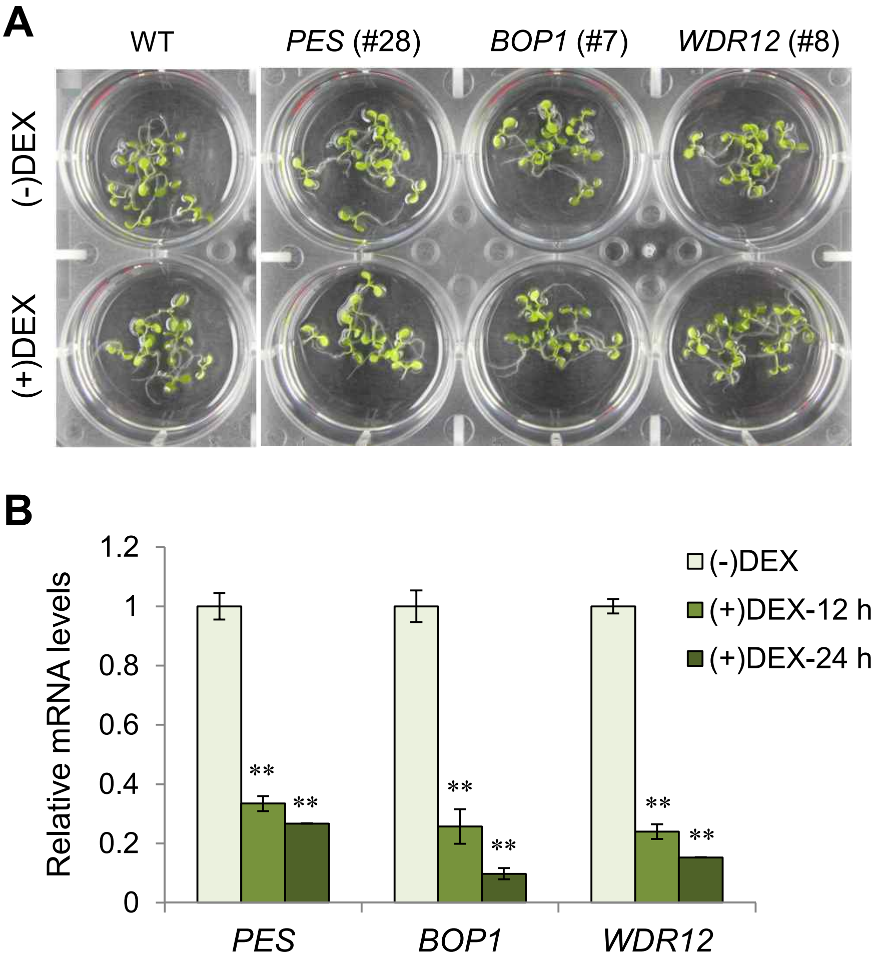

Supplementary Fig. S5. Gene silencing in the RNAi seedlings grown in liquid culture.

(A) *PES* (#28), *BOP1* (#7), and *WDR12* (#8) RNAi seedlings (7 DAS) grown in liquid culture were treated with ethanol (-) or 20 μM DEX (+) for 24 h.

(B) Real-time quantitative RT-PCR analyses of gene silencing. The RNAi seedlings grown in liquid culture were treated with ethanol (-) or 20 μM DEX (+) for 12 h or 24 h. The first leaves were collected for the analyses. *PES*, *BOP1*, and *WDR12* transcript levels were quantified relative to (-)DEX samples using *UBC10* mRNA levels as a control. Each value represents the mean ± SD of three replicates per experiment. *, $P \leq 0.05$; **, $P \leq 0.01$.

Supplementary Figure S6

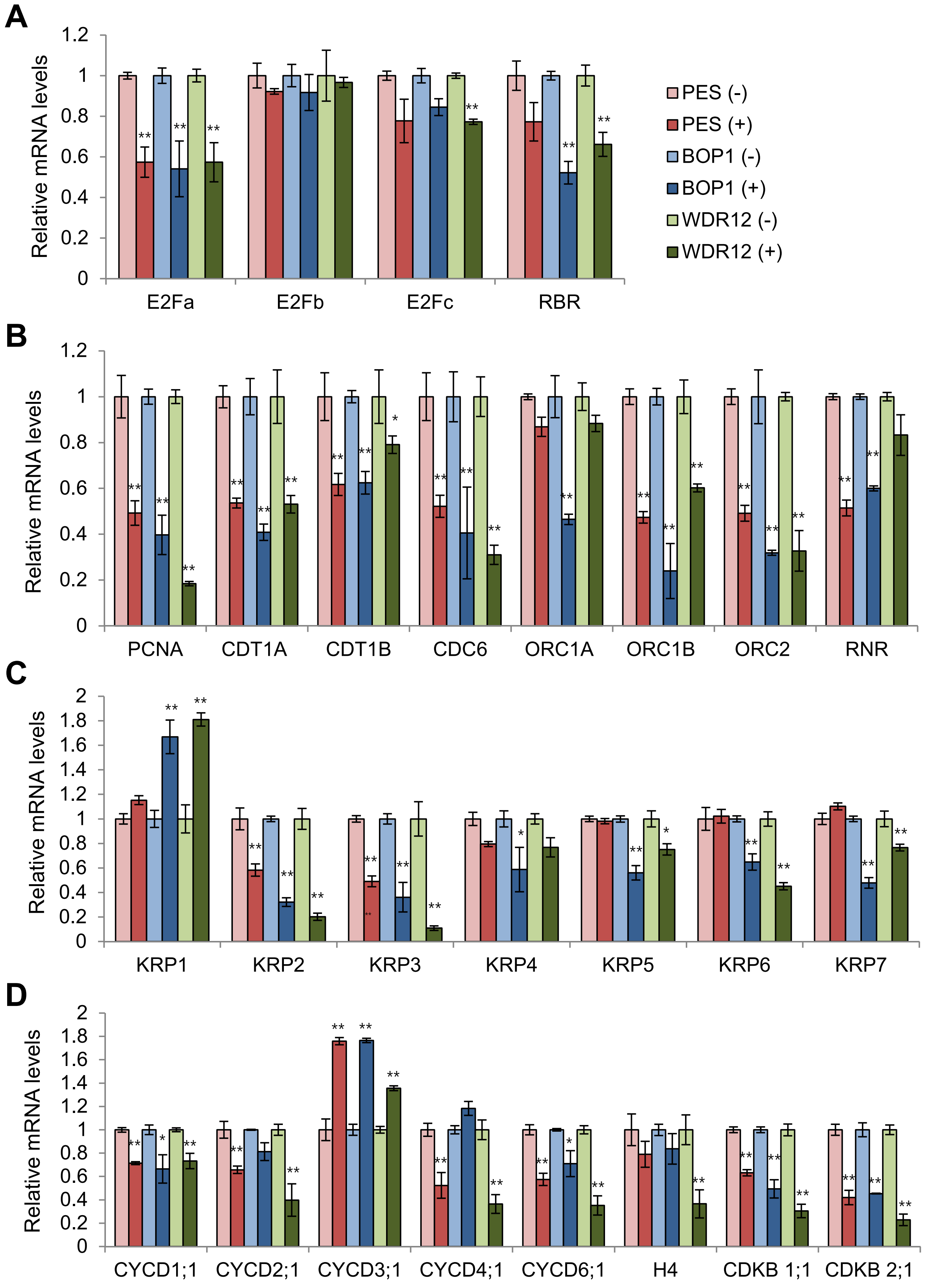

Supplementary Fig. S6. Real-time quantitative RT-PCR analyses for the expression of cell cycle-related genes after 12-h DEX treatment.

The RNAi seedlings (7 DAS) grown in liquid culture were treated with ethanol (-) or 20 μM DEX (+) for 12 h. The first leaves were collected for the analyses. Transcript levels were quantified relative to (-)DEX samples using *UBC10* mRNA levels as a control. Each value represents the mean ± SD of three replicates per experiment. *, $P \leq 0.05$; **, $P \leq 0.01$.

(A) E2F/RBR pathway genes.

(B) S-phase genes.

(C) KRP family genes.

(D) CycD family, histone H4, and CDKB genes.

Supplementary Figure S7

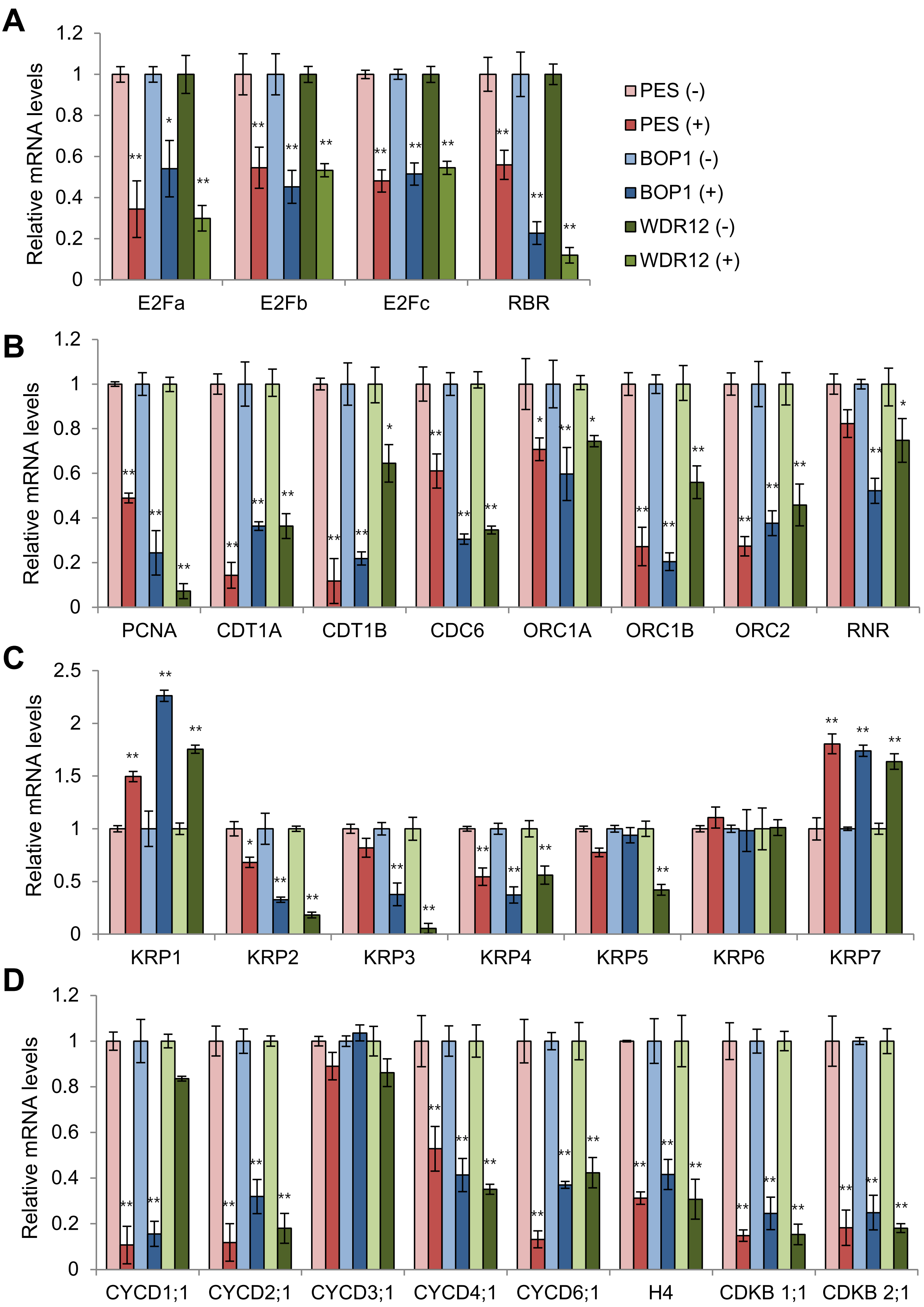

Supplementary Fig. S7. Real-time quantitative RT-PCR analyses for the expression of cell cycle-related genes after 24-h DEX treatment.

The RNAi seedlings (7 DAS) grown in liquid culture were treated with ethanol (-) or 20 μM DEX (+) for 24 h. The first leaves were collected for the analyses. Transcript levels were quantified relative to (-)DEX samples using *UBC10* mRNA levels as a control. Each value represents the mean ± SD of three replicates per experiment. *, $P \leq 0.05$; **, $P \leq 0.01$.

(A) E2F/RBR pathway genes.

(B) S-phase genes.

(C) KRP family genes.

(D) CycD family, histone H4, and CDKB genes.

Supplementary Figure S8

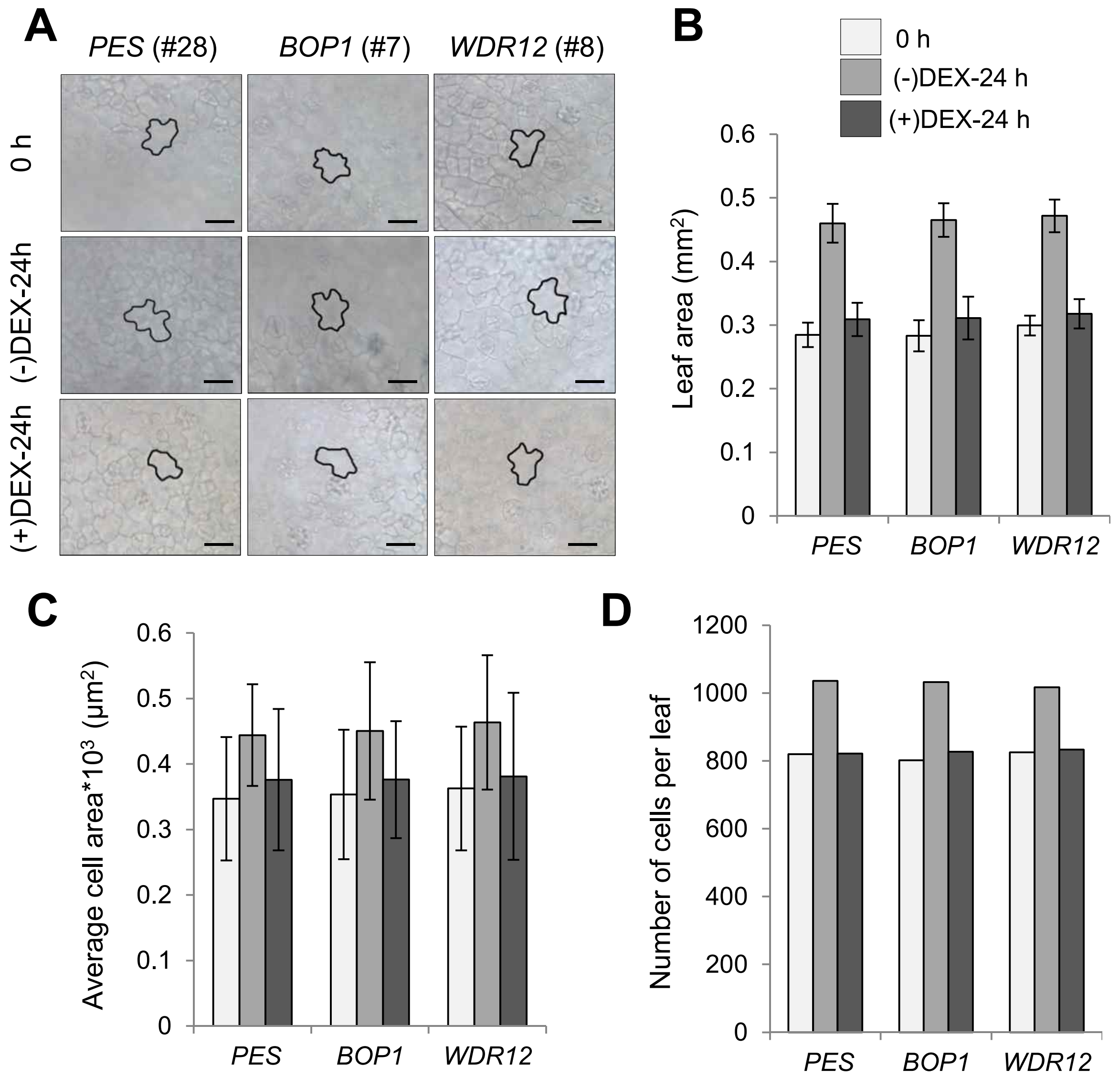

Supplementary Fig. S8. Leaf cell division and expansion after 24-h DEX treatment.

The RNAi seedlings (7 DAS) grown in liquid culture were treated with ethanol (-) or 20 μM DEX (+) for 24 h, and the first leaves were collected for the analyses.

(A) Representative abaxial epidermal cells of the first leaves. Individual cells are visualized by *black outlines* using ImageJ. Scale bars = 20 μm.

(B) Average leaf area.

(C) Average leaf epidermal cell area.

(D) Calculated number of epidermal cells per leaf.

Supplementary Figure S9

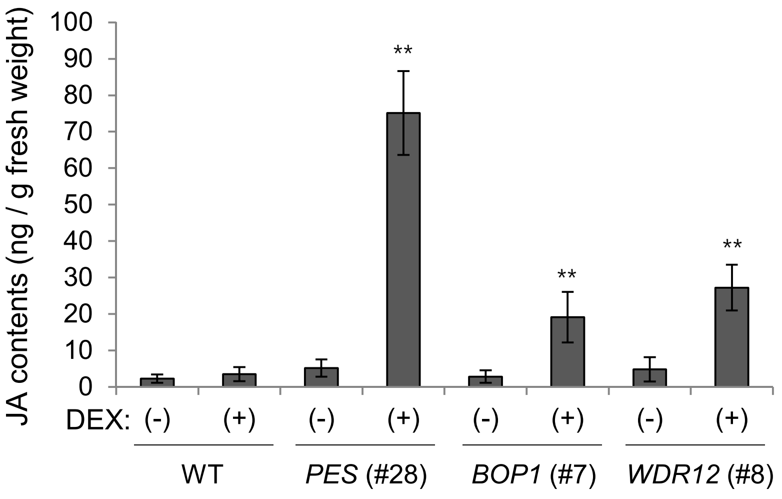

Supplementary Fig. S9. Endogenous JA contents of the RNAi seedlings.

For the measurement the RNAi seedlings were grown in MS media for 2 weeks, and then transferred to MS media containing ethanol (-DEX) or 5 μM DEX for further growth for 6 days. Each value represents the mean ± SD of three replicates per experiment. *, $P \leq 0.05$; **, $P \leq 0.01$.
